# Hormonal Contraceptives and Depression: A Proteomic Analysis Using Neuronal Models

**DOI:** 10.1002/prca.70017

**Published:** 2025-09-10

**Authors:** Sam Thilmany, Andreas Thomas, Yvonne Reinders, Farhad Shakeri, Matthias Vogel, Albert Sickmann, Catharina Scholl, Mario Thevis

**Affiliations:** ^1^ Institute of Biochemistry Center for Preventive Doping Research German Sport University Cologne Cologne Germany; ^2^ Federal Institute for Drugs and Medical Devices Bonn Germany; ^3^ Leibniz‐Institut für Analytische Wissenschaften – ISAS – e.V. Dortmund Germany; ^4^ Core Unit for Bioinformatics Data Analysis Medical Faculty University of Bonn Bonn Germany; ^5^ European Monitoring Center for Emerging Doping Agents (EuMoCEDA) Cologne, Bonn Germany

**Keywords:** hormonal contraception, major depressive disorder, neuronal cell culture, oral contraception, quantitative proteomics

## Abstract

**Purpose:**

Hormonal contraceptives are linked to a higher prevalence of depressive symptoms. Given their popularity in Western countries, understanding the biochemical effects on neuronal cells is crucial to minimizing mental health risks.

**Experimental Design:**

Neural progenitor cells were treated with ethinyl estradiol (EE) and levonorgestrel (LNG), two synthetic sex hormones commonly used in oral contraception, and S‐23, a selective androgen receptor modulator developed as a potential synthetic sex hormone for male hormonal contraception. Label‐based quantitative proteomics with the TMTpro 16plex tandem mass tags were used to assess protein expression changes between treated and untreated cells.

**Results:**

Treatment of human neural progenitor cells with EE, LNG, EE + LNG, and S‐23 led to distinct and overlapping proteomic changes, with enrichment in pathways related to inflammation, oxidative stress, transcriptional regulation, and cell death. Disease association analyses linked these changes to neurodegenerative and psychiatric conditions, including mechanisms relevant to depression.

**Conclusions and Clinical Relevance:**

These findings suggest that hormonal compounds used in contraception and performance enhancement may influence molecular pathways implicated in mental health, particularly depression. Although not directly translatable to clinical outcomes, the results support the need for further investigation into the neuropsychiatric effects of hormonal treatments.

**Summary:**

- This study addresses a pressing clinical need to better understand the potential mental health impacts of widely used hormonal contraceptives. While highly effective for pregnancy prevention, compounds such as ethinyl estradiol and levonorgestrel have repeatedly been associated with increased risk of depressive symptoms, highlighting the importance of investigating their molecular effects on neural systems.
- To explore this, we applied label‐based quantitative proteomics in an undifferentiated human neural progenitor cell model treated with ethinyl estradiol, levonorgestrel, their combination, and the selective androgen receptor modulator S‐23. The treatments induced distinct and overlapping changes in protein expression, with enrichment in pathways related to inflammation, oxidative stress, cell adhesion, chromatin dynamics, and programmed cell death—biological processes known to intersect with mechanisms implicated in depression.
- These findings offer insight into how synthetic hormones and hormone‐like compounds may modulate neuronal biology, potentially contributing to adverse mental health outcomes. However, due to limitations of the in vitro model—such as the absence of systemic context, pharmacokinetics, and mature neuronal function—these results are primarily hypothesis‐generating. They underscore the importance of further research to clarify the pathophysiological mechanisms linking hormonal treatments to affective disorders and to better assess the mental health risks of these compounds.

**Abbreviations**
AFT: accelerated failure time
AGC: automatic gain control
BCA: bicinchoninic acid
COC: combined oral contraceptives
DAP: differentially abundant proteins
DDA: data‐dependent acquisition
DOSE: disease ontology semantic and enrichment analysis
DPBS: Dulbecco's phosphate buffered saline
DTT: dithiothreitol
EE: ethinyl estradiol
FA: formic acid
FC: fold‐change
FDR: false discovery rate
FWHM: full width at half maximum
GO: gene ontology
HR: high resolution
IAA: iodoacetamide
KEGG: Kyoto Encyclopedia of Genes and Genomes
LBQ: label‐based quantification
LFQ: label‐free quantification
LNG: levonorgestrel
NCE: normalized collision energy
PSM: peptide‐spectrum match
RII: reporter ion intensity
SARM: selective androgen receptor modulator
TMT: tandem mass tag
UPLC: ultra‐performance liquid chromatography
WADA: World Anti‐Doping Agency

## 1 Introduction

The advent of hormonal contraception has been one of the most transformative developments in women's health over the past century. Since their introduction in the 1960s, combined oral contraceptives (COCs) have provided women with unprecedented control over their reproductive health, contributing significantly to women's liberation and self‐determination [1]. The widespread adoption of COCs has led to profound societal changes, enabling women to pursue higher education, establish careers, and make more autonomous decisions about their personal lives [2]. The health benefits of COCs extend beyond pregnancy prevention; they are also employed, among other conditions, in the management of menstrual disorders [3], endometriosis [4], and acne [5]. Despite these substantial advantages, the impact of COCs on mental health, particularly their potential role in the development of depression, remains a contentious issue within the scientific community. In addition to those substances that are used as contraceptives for women, also substances that were originally developed as male contraceptives but are now misused in sport [6] warrant consideration. Thus, the investigation of the potential influence of synthetic sex hormones on mood is also relevant in the context of doping prevention. After all, synthetic sex hormones are still among the most commonly abused substances in sport [6] and are readily associated with aggressive mood in athletes [7].

Depression is one of the most common causes of disability worldwide [8], significantly impacting individuals, society, and healthcare systems [9]. The incidence of depressive disorders in women is 1.5 times higher than in men [10]. Moreover, the percentage of the total number of disability‐adjusted life years (DALY) attributable to depressive disorders is 1.8 times higher in women than in men [10], indicating that depression not only affects women more frequently but also leads to a greater overall burden of disability in women compared to men. The relationship between hormonal contraception and mood changes has been extensively debated, with studies yielding controversial results [11]. Epidemiological studies suggest an association between COC use and an increased risk of developing depression [12, 13], but these findings are not uniformly supported across the literature [14, 15]. The retrospective observational nature of many epidemiological studies introduces healthy user bias [16], wherein individuals who experience adverse effects may discontinue use and thus not be represented in long‐term studies. Additionally, studies relying on patient questionnaires are susceptible to recall bias [17], as participants’ recollections of mood changes may be influenced by their current mental state or external factors.

Both observational studies and patient questionnaires also grapple with selection bias and numerous confounding factors, such as pre‐existing mental health conditions, lifestyle variables, and socioeconomic status [18, 19]. These limitations underscore the need for alternative approaches to investigating the potential neuropsychiatric effects of synthetic sex hormones. Although some researchers have posited that synthetic hormones in COCs could influence mood by altering neurotransmitter systems, inflammatory pathways, or neuroplasticity, the precise biological mechanisms remain poorly understood [20].

By employing an in vitro cell culture model to investigate the biochemical effects of synthetic sex hormones on neuronal cells, the present research project addresses these gaps. The ReNcell VM Human Neural Progenitor Cell Line was used throughout this study. This cell line is derived from the ventral mesencephalic region of the human fetal brain. The ReNcell VM cells are multipotent neuronal progenitor cells capable of differentiating into neurons and glial cells, making them an ideal model for studying neural development and neurodegenerative diseases. The cells were treated with ethinyl estradiol (EE) and levonorgestrel (LNG), both of which are widely used in hormonal contraception [21].

Additionally, S‐23, a selective androgen receptor modulator (SARM) developed as a potential male contraceptive [22], was used as a test compound. Unlike EE and LNG, which are approved drugs, S‐23 is not approved for clinical use. Due to its potential for abuse for doping purposes, S‐23 is on the World Anti‐Doping Agency's (WADA) Prohibited List [23].

In this study, quantitative proteomics was utilized to analyze protein expression changes in treated versus untreated neuronal cells. To this end, the proteins were extracted from the treated cells, digested, and analyzed using nano LC‐high resolution (HR)‐MS. For the functional analysis, Gene Ontology (GO) term enrichment analysis, Kyoto Encyclopedia of Genes and Genomes (KEGG) pathway enrichment analysis, Disease Ontology Semantic and Enrichment analysis (DOSE), and disease enrichment analysis using DisGeNET's curated database were performed. Although DOSE leverages structured ontologies to identify associations between gene sets and disease terms within a hierarchical framework, DisGeNET provides a complementary perspective by integrating expert‐curated and literature‐based gene‐disease associations, offering broader coverage of disease‐related genes. Using these methods, this study aimed to investigate whether neuronal cells exhibit changes associated with affective disorders as a result of treatment with different synthetic sex hormones and could thus provide an indication of whether a link exists between the intake of these substances and the development of mood disorders.

## 2 Materials and Methods

Detailed information on the consumables and devices, including product names, numbers, brand names, and suppliers, is provided in Tables S1 and S2 of the Supporting Information.

### 2.1 Cell Culture

In this experiment, the multipotent neuronal progenitor cells ReNcell VM were maintained in an undifferentiated state. Cells were cultured according to the manufacturer's protocol with minor modifications. In brief, a pre‐culture phase was initiated by seeding cells into laminin‐coated 182 cm^2^ flasks and culturing them until they reached approximately 90% confluency to ensure an adequate cell supply for subsequent experiments. Cell detachment was performed using an enzyme‐free cell dissociation buffer, and cell counting was conducted using the Countess 3 Automatic Cell Counter.

For the treatment phase, cells were plated into 16 laminin‐coated 25 cm^2^ flasks. Three flasks were allocated for each of the following treatments: 100 ng/mL EE, 100 ng/mL LNG, a combination of 100 ng/mL EE and 100 ng/mL LNG, and 100 ng/mL S‐23. All substances were first dissolved in DMSO to form stock solutions, which were then added to the cell culture medium, resulting in a final DMSO concentration of 100 ppm in the cell culture medium. Additionally, two flasks were treated with 100 ppm DMSO as controls, and two flasks received only the complete growth medium. This results in three pseudo‐biological treatment replicates for the four treatments and two pseudo‐biological treatment replicates for the two controls.

During the 14‐day treatment, cultures were monitored regularly under an inverted microscope, with no morphological changes observed during these checks. Medium refreshment was performed every other day. Cell passaging was performed upon reaching approximately 90% confluency. Cell viability was assessed during sub‐culturing and at the final harvesting step using Trypan blue staining. The determined values remained above 85% for all samples and at every harvesting step.

At the end of the treatment period, cells were harvested using the enzyme‐free cell dissociation buffer, counted, and aliquoted into 10^6^‐cell aliquots. The aliquots were washed with Dulbecco's PBS (DPBS), pelleted (5 min, 300 × *g*), and the supernatant was removed. They were then stored at −80°C until further processing.

### 2.2 Label‐Based Quantification (LBQ) of Shotgun Proteomics

The sample preparation and the mass spectrometric analysis were carried out at the Institute of Biochemistry of the German Sport University Cologne, Cologne, Germany.

#### 2.2.1 Sample Preparation

Protein extraction, reduction, alkylation, Trypsin/Lys‐C digestion, and cleanup were conducted using the EasyPep MS Sample Prep Kit, strictly adhering to the manufacturer's protocol. Briefly, cells underwent lysis, and protein concentrations were determined using a bicinchoninic acid (BCA) assay. Following the manufacturer's instructions, 100 µg of protein were subjected to reduction and alkylation using iodoacetamide (IAA). Subsequently, the proteins were enzymatically digested with Trypsin/LysC, and the resulting peptides were purified. The purified peptides were labeled with 0.5 mg TMTpro 16plex label reagent, following the EasyPep protocol, and quantified using the NanoDrop One spectrophotometer. The samples were then pooled, ensuring even distribution across the 16 tandem mass tag (TMT) channels. Finally, the pooled sample was aliquoted into aliquots with a total peptide content of 100 µg each and stored at −80°C until further processing.

A pooled sample aliquot underwent desalting and pre‐fractionation utilizing the High pH Reversed‐Phase Peptide Fractionation Kit prior to analysis.

#### 2.2.2 LC‐HR‐MS/MS Data Acquisition

For chromatographic separation, eluent A, composed of 0.1% formic acid (FA) in ULC/MS water, and eluent B, consisting of 80% ULC/MS ACN with 0.1% FA, were utilized.

For each injection, 1 µg of peptide fraction was loaded onto the PepMap Neo Trap Cartridge (C18, 100 Å, 5 µm, 300 µm × 5 mm) trapping column using eluent A. Peptides were trapped for 5 min at a flow rate of 3 µL/min using a nanoACQUITY ultra‐performance liquid chromatography (UPLC) system operated in single‐pump trapping mode.

Analytical separation was performed at 300 nL/min on an in‐house‐prepared integrated emitter column (ReproSil‐Pur 120 C18‐AQ, 120 Å, 3 µm, 75 µm × 50 cm). A gradient from 0% eluent B to 50% eluent B over 180 min was employed, followed by a 15 min wash with 100% eluent B and a subsequent re‐equilibration step for 15 min at 0% eluent B.

Peptides eluted from the analytical column were introduced via a Nanospray Flex Ion Source into the Q Exactive mass spectrometer. The following instrument settings were applied: spray voltage of 4.5 kV, capillary temperature of 250°C, and S‐Lens level of 65.

LC‐HR‐MS/MS data were acquired in a Top‐15 data‐dependent acquisition (DDA) mode. Full scan parameters included a resolving power of 70,000 full width at half maximum (FWHM) at *m*/*z *200, an automatic gain control (AGC) target of 3 × 10^6^ ions, a maximum injection time of 50 ms, and a scan range of *m*/*z* 375–1500. MS/MS data acquisition settings included a resolving power of 35,000 FWHM at *m*/*z* 200, an AGC target of 2 × 10^5^ ions, a maximum injection time of 250 ms, an isolation width of *m*/*z* 0.7, a stepped normalized collision energy (NCE) of 32% and 34%, the inclusion of ions with charge states 2–7, isotopes exclusion, preferred peptide match, and a dynamic exclusion window of 45 s.

### 2.3 Label‐Free Quantification (LFQ) of Shotgun Proteomics

The sample preparation and mass spectrometric analysis were conducted by the Department of Bioanalytics at the Leibniz‐Institut für Analytische Wissenschaften—ISAS—e.V., Dortmund, Germany.

#### 2.3.1 Sample Preparation

A total of 100 µg of solubilized proteins was reduced in 10 mM dithiothreitol (DTT) at 56°C for 30 min and subsequently alkylated in 30 mM IAA at RT for 30 min in the dark. Subsequently, the lysate was digested using the S‐Trap mini procedure according to the manufacturer's protocol.

#### 2.3.2 LC‐HR‐MS/MS Data Acquisition

Trypsin‐digested proteins were dissolved in 0.1% (v/v) TFA and analyzed by nano LC‐HR‐MS/MS using 1 µg of material per injection. Samples were loaded on an Ultimate 3000 Rapid Separation LC coupled to a Q Exactive HF Orbitrap mass spectrometer. For chromatography, a reversed‐phase column (Acclaim C18 PepMap 100, 100 Å, 3 µm, 75 µm × 50 cm) was used (linear increase of solvent B [84% ACN with 0.1% FA] to 35% for 120 min). MS scans were acquired using the following settings: The mass spectrometer was operated in DDA mode with full MS scans from *m*/*z* 300 to 1500 at a resolution of 60,000 FWHM at *m*/*z* 200. The AGC was set to 3 × 10^6^ ions with a maximum injection time of 120 ms. Following each survey scan, the most intense ions above a threshold ion count of 5 × 10^4^ ions were selected for collision‐induced dissociation at an NCE of 27%. Dynamic exclusion was set to 12 s. Product ions were acquired at a resolution of 15,000 FWHM at *m*/*z* 200, with an AGC of 5 × 10^4^ ions and a maximum injection time of 50 ms.

### 2.4 Data Analysis

Raw mass spectrometry data were processed using the Proteome Discoverer software (version 3.0.1.27). Spectra were searched against the UniProtKB/Swiss‐Prot *Homo sapiens* protein database (Taxon ID: 9606; Version 2022‐08‐03) using the Sequest HT search engine with Trypsin (Full) as the proteolytic enzyme, allowing up to two missed cleavages. Precursor and fragment mass tolerances were set to 10 ppm and 0.02 Da, respectively. Static modifications included carbamidomethylation of cysteine (+ 57.021 Da) for both LFQ and TMT workflows, and TMTpro labeling of lysine residues and peptide N‐termini (+ 304.207 Da) for TMT samples. The oxidation of methionine (+ 15.995 Da) was set as a dynamic modification. Peptide‐spectrum matches (PSMs) were validated using Percolator with a strict target false discovery rate (FDR) of 1% and a relaxed FDR of 5%. For TMT‐based samples, quantification was performed using the Reporter Ions Quantifier node with an integration tolerance of 20 ppm and the Most Confident Centroid integration model. Additional feature detection for LFQ was performed using the Minora Feature Detector with a minimum trace length of five and a signal‐to‐noise threshold of one.

The resulting lists of PSMs were exported as text files and used as input for downstream analysis in R (version 4.5.0). The R analysis pipeline is available on GitHub (https://github.com/SamThilmany/ILLUMINE‐202401_Analysis‐Pipeline). The MSstats (version 4.16.0) [24] and MSstatsTMT (version 2.16.0) [25] packages were used to convert, normalize, and statistically model the LFQ and LBQ data, respectively. The LFQ dataset was primarily used for the qualitative validation of the LBQ data, confirming the reproducibility and reliability of protein identifications and abundance patterns across both quantification methods.

Multiple quality control assessments were performed throughout the analysis. These included the visualization of missing values to assess data completeness, followed by filtering proteins with excessive missingness. The dynamic range of quantified proteins was evaluated to confirm appropriate signal detection across the experimental conditions. Labeling efficiency was assessed to verify the quality and consistency of TMT labeling in the LBQ dataset. Additionally, the effectiveness of normalization procedures was assessed through visualization of the abundance distribution.

Group comparisons between treatments and controls were used to identify differentially abundant proteins (DAPs) based on empirical Bayes moderated linear models. The Pearson correlation coefficient between LBQ and LFQ fold changes was taken as a measure for the reproducibility and validity of the LBQ dataset.

Subsequent functional analysis focused on the LBQ dataset. Proteins that passed defined FDR thresholds were subjected to enrichment analyses. These analyses included GO term enrichment across biological process categories and KEGG pathway enrichment using the clusterProfiler package (version 4.16.0) [26], as well as disease enrichment based on the disease ontology (DO) and DisGeNET [27] using the DOSE package (version 4.2.0) [28].

## 3 Results and Discussion

### 3.1 Technical Analysis

Labeling efficiency using TMTpro 16plex reagents reached 99.79%, with exceptionally uniform distribution across all 16 channels, as indicated by the ratios of individual channel reporter ion intensities (RII) to the mean RII of the entire TMT set [29], as shown in the density plots in Figure S1. This high labeling efficiency and uniformity represent ideal starting conditions for downstream quantitative analysis, minimizing variability introduced at the sample preparation stage and ensuring comparability across samples [29].

The identification rate in the LBQ dataset (88.6%) surpassed that of the LFQ dataset (63.9%) by 24.7 percentage points. This substantial improvement can be attributed to several technical advantages inherent to the LBQ workflow, including the use of offline fractionation and an extended linear gradient in LC‐MS. These steps reduce sample complexity at any given time point, mitigating the effects of co‐isolation and chimeric spectra. As a result, spectral clarity improves, thereby enhancing the identification rates of peptides and proteins. This is also reflected in the final protein identification counts, with LBQ yielding 7109 proteins compared to 1858 in LFQ.

Despite this difference in depth, there was considerable concordance between the two methods: 1785 proteins (96.1% of those identified in LFQ) were also detected in LBQ. This high overlap suggests that the core proteomic signature remains consistent across quantification strategies. Moreover, when proteins were stratified by treatment contrast, this intersection remained dominant (Figure S5), highlighting the robustness of the findings regardless of the quantification approach used.

A known limitation of LFQ is its susceptibility to missing values [30, 31], which was clearly observed in our dataset. LFQ PSM‐level data exhibited 32.4% missingness, whereas LBQ data had a near‐complete coverage with only 0.01% missing values (Figure S2). This difference is not merely technical but has significant downstream implications. High missingness in LFQ often necessitates imputation, which can introduce bias, especially if missing values are not missing at random. This effect is evident in Figures S7 and 2, where LFQ abundance data appear skewed to the left—particularly in the LNG and EE + LNG versus DMSO contrasts, with mean log_2_ fold changes of −0.35 and −0.30, respectively. This skew likely results from the imputation of missing values with artificially low abundance estimates, leading to systematically deflated protein quantifications. Specifically, the imputation method used by MSstats is based on an accelerated failure time (AFT) model, which assumes that all missing values are not missing at random but rather are censored due to signal intensities falling below a detection threshold [24]. Although this assumption is appropriate for missing values caused by true low abundance, it can lead to biased results when data are missing for other reasons—such as stochastic sampling effects inherent in DDA. In such cases, the AFT model will incorrectly impute randomly missing values with low‐intensity estimates, thereby artificially depressing the calculated protein abundances and introducing a systematic leftward skew in the data distribution.

Prior to protein‐level summarization and data normalization, peptides were grouped by protein. Proteins with ≥50% missing values across samples (LFQ) or TMT channels (LBQ) were excluded. This filtering step removed 952 proteins from the LFQ dataset, while no proteins were excluded from the LBQ dataset. As shown in Figure S3, the filtered LFQ dataset now comprised 313,232 peptide ion entries with a reduced overall missingness of 29.26%. Per‐sample missingness ranged from 22.19% to 42.74%, compared to previously 25.17% to 45.75% before filtering.

**FIGURE 1:**
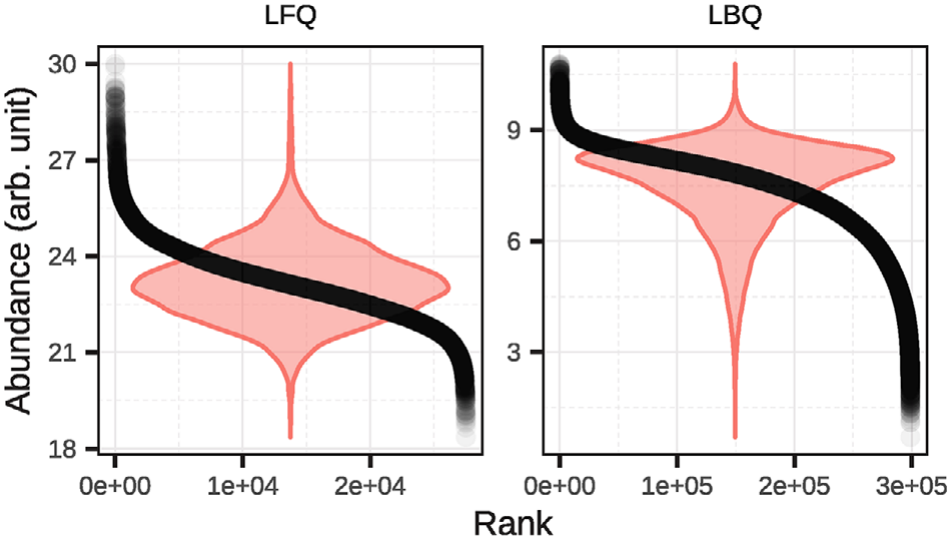
Dynamic range of protein abundances in label‐free (LFQ) and label‐based (LBQ) proteomics datasets. Protein abundance values (y‐axis) are plotted against ranked protein identifiers (x‐axis), ordered by decreasing abundance. The scatter plot illustrates the characteristic sigmoidal distribution of protein abundances, with both quantification strategies capturing a wide dynamic range. The LFQ data (left) and LBQ data (right) show a steep initial decline in abundance for highly expressed proteins, followed by a gradual linear region and a second drop for low‐abundance proteins. The LBQ curve displays a slightly skewed tail, suggesting altered quantification performance in the lower abundance range. The violin plot in the background visualizes the number of proteins present at each abundance level.

Protein‐level summarization was followed by data normalization. As illustrated in Figure S4, normalization resulted in well‐aligned abundance distributions across all samples and TMT channels. After summarization, 1858 unique proteins were quantified in the LFQ dataset, and 7109 in the LBQ dataset, with 96.07% of the LFQ‐identified proteins also present in the LBQ dataset. Please refer to Figure S5 for an overview of the overlaps per contrast. Evaluation of the dynamic range revealed a similarly broad overall distribution of protein abundances in the LFQ dataset (∆ = 11.63) and the LBQ dataset (∆ = 10.08), as shown in Figure 1. The spread between the 5% quantile and the 95% quantile was also very comparable between the two methods, with ∆ = 3.79 for LFQ and ∆ = 3.72 for LBQ.

**FIGURE 2:**
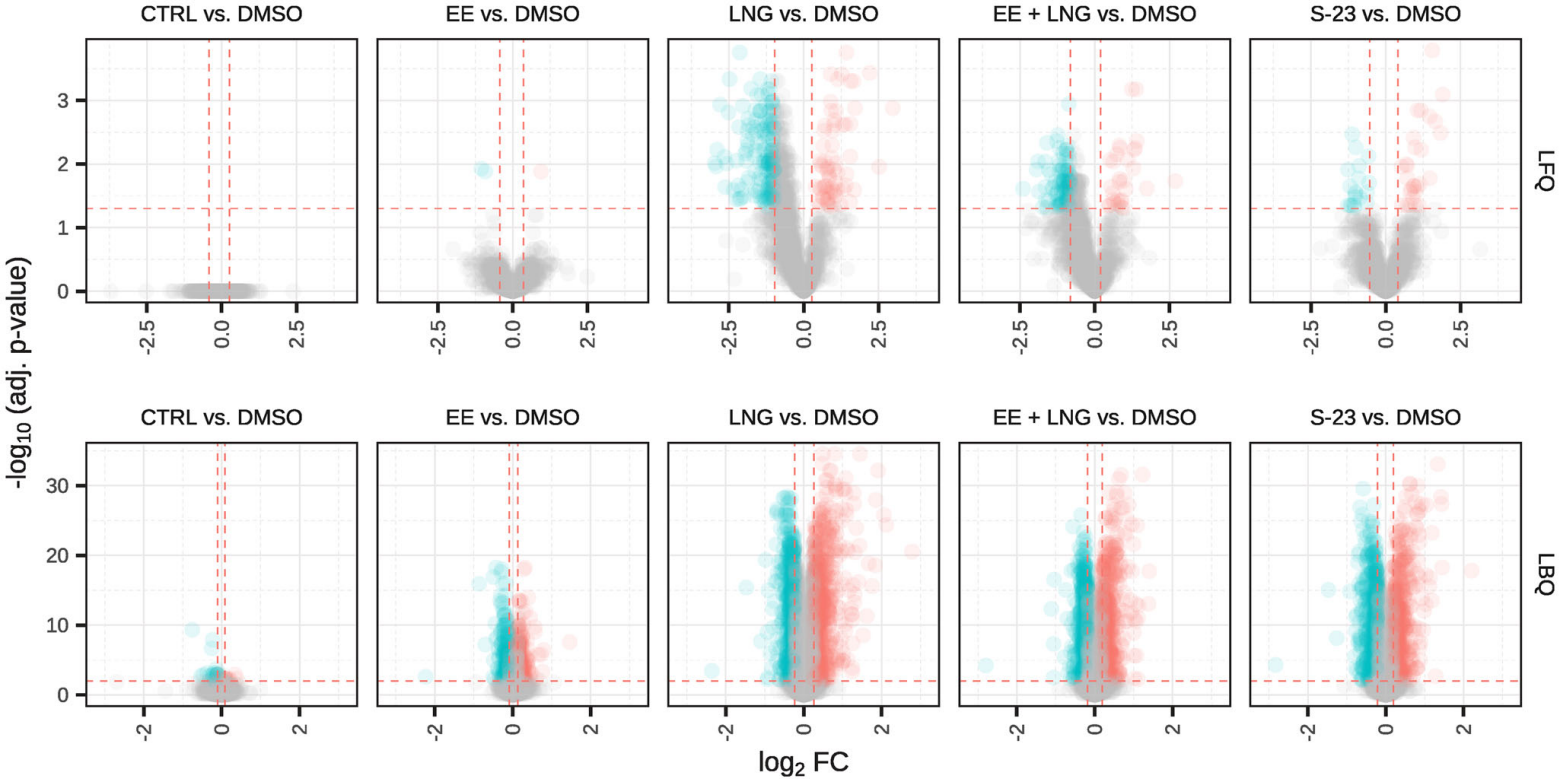
Differential abundance analysis of proteins in response to treatments. Volcano plots visualize the DAPs identified in pairwise comparisons of each treatment group versus the DMSO control. The top row displays results from the LFQ dataset, and the bottom row shows results from the TMT‐based LBQ dataset. Columns represent individual treatments. The x‐axis denotes log_2_ FC, and the y‐axis shows the −log₁₀ of the adjusted *p* values. Proteins significantly upregulated in the treatment group are colored red, significantly downregulated proteins are colored blue, and non‐significant proteins are shown in gray. Significance thresholds (FDR < 0.05 for LFQ and FDR < 0.01 for LBQ) and log_2_ FC thresholds are indicated by the red dashed lines.

Differential protein abundance analysis was performed to compare each treatment group against the DMSO control using both quantification methods. Volcano plots (Figure 2) display log_2_ fold change (FC) versus −log₁₀ (adjusted *p* value) with significantly up‐ and down‐regulated proteins colored in red and blue, respectively, and non‐significant proteins in gray. Despite the dynamic range being very similar, the LFQ data exhibited a higher standard deviation and, therefore, a greater spread of log_2_ FC values across all comparisons compared to the LBQ data. This effect is observable in Figures 1 and 2, as well as in Figure S7. In the LFQ dataset, 0.17% of proteins were significantly differentially abundant in the EE versus DMSO comparison, 27.79% in the LNG versus DMSO comparison, 12.94% in the EE + LNG versus DMSO comparison, and 2.59% in the S‐23 versus DMSO comparison (FDR < 0.05). No significant differential abundance was observed in the CTRL versus DMSO comparison. In the LBQ dataset, significant DAPs (FDR < 0.01) were detected in all contrasts: 15.68% in EE versus DMSO, 54.59% in LNG versus DMSO, 45.89% in EE + LNG versus DMSO, 45.32% in S‐23 versus DMSO, and 0.59% in CTRL versus DMSO. Summary statistics for significant and non‐significant DAPs across all contrasts are provided in Figure S6. To refine the classification of DAPs, an additional fold change threshold was applied based on the distribution of log_2_ FC values within each contrast, as illustrated in Figure S7. Following *p* value‐based significance filtering (red dashed horizontal line in Figure 2), proteins were classified as significantly upregulated if their log_2_ FC exceeded (mean + standard deviation) or downregulated if below (mean—standard deviation) for the respective contrast (red dashed vertical lines in Figure 2).

In the LFQ dataset, the application of these thresholds for *p* value and FC, removed all DAPs in the CTRL versus DMSO contrast and retained three DAPs in EE versus DMSO, 227 DAPs in LNG versus DMSO, 132 in EE + LNG versus DMSO, and 47 in S‐23 versus DMSO. In the LBQ dataset, 30 remained for CTRL versus DMSO, 532 for EE versus DMSO, 1356 for LNG versus DMSO, 1296 for EE + LNG versus DMSO, and 1209 for S‐23 versus DMSO.

To confirm that the findings in the LBQ dataset are biologically meaningful and not artifacts of the quantification strategy, we cross‐validated the results using the independently acquired LFQ dataset. Although both approaches are based on bottom‐up mass spectrometry, they differ in several key aspects, including sample preparation workflows, quantification methods (isobaric labeling vs. label‐free), instrumentation, laboratory personnel, and data processing pipelines. These differences help reduce shared sources of technical bias and provide an additional layer of confidence in the biological validity of the observed changes in protein abundance.

**FIGURE 3:**
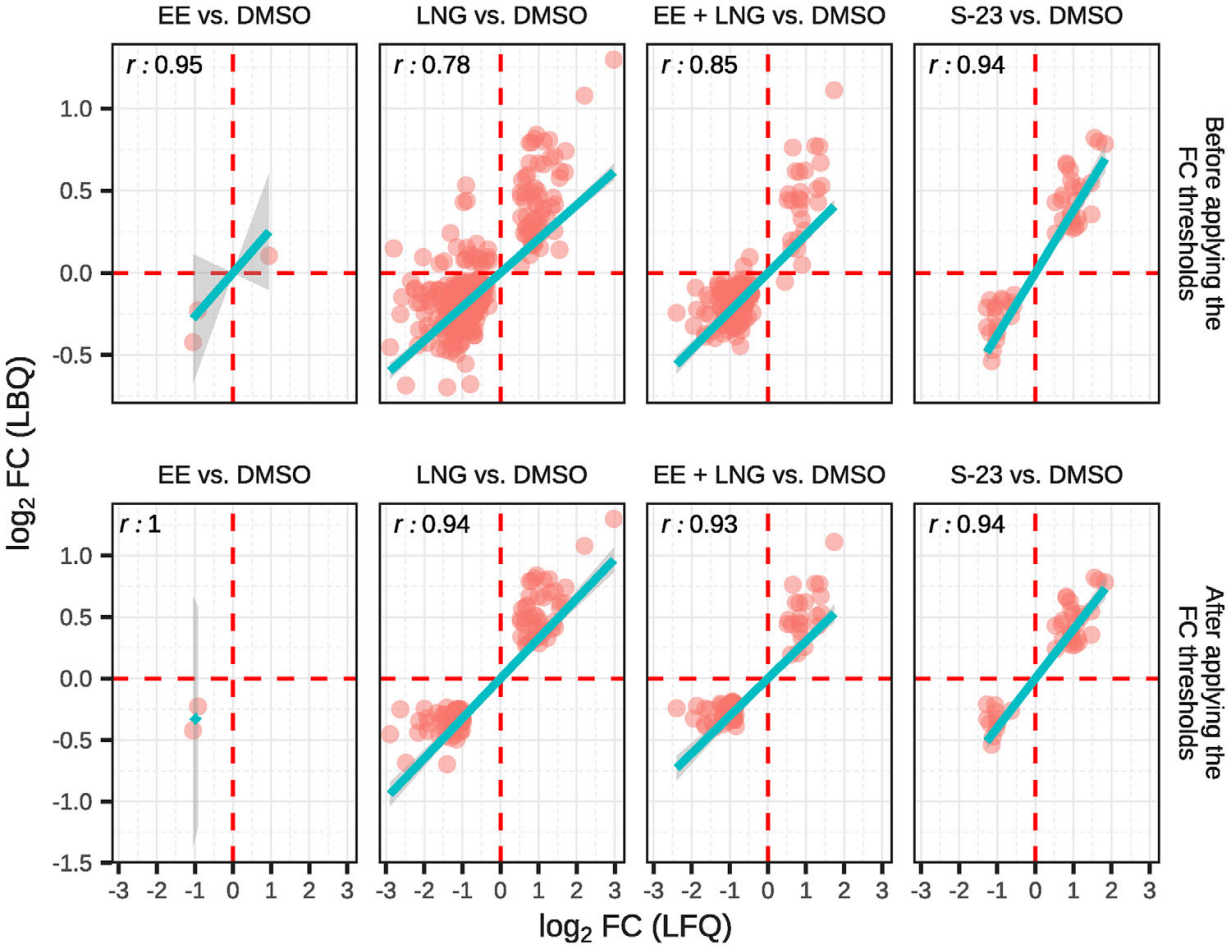
Correlation of FC estimates between LFQ and LBQ quantification. Scatter plots showing the comparison of log_2_ FC values for DAPs identified in both the LFQ and LBQ datasets for the four treatment contrasts (EE, LNG, EE + LNG, and S‐23 vs. DMSO). Each point represents a protein, plotted with the LFQ's log_2_ FC on the x‐axis and LBQ's log_2_ FC on the y‐axis. The top panel includes proteins that passed the *p* value threshold only (FDR < 0.05 for LFQ and < 0.01 for LBQ), while the bottom panel includes proteins that also passed the log_2_ FC threshold (outside the mean ± standard deviation range). Red dashed lines indicate zero change, and blue lines show the linear regression fit, which was forced through the origin. Pearson correlation coefficients (*r*) are displayed in each facet.

Remarkably, the agreement between the datasets was strong. Among proteins meeting the FDR threshold (1% for LBQ, 5% for LFQ), Pearson correlation coefficients ranged from 0.78 to 0.95 across treatment contrasts, as illustrated in Figure 3. When further restricted to proteins exceeding contrast‐specific FC thresholds, correlations improved even more, ranging from 0.93 to 0.94—and reaching 1.0 in the EE versus DMSO contrast, albeit with only two proteins. This level of concordance, despite methodological differences and analytical constraints, provides compelling evidence that the observed trends in protein abundance are biologically relevant and not an artifact of the quantification strategy used.

In sum, the LBQ approach not only provided deeper proteome coverage and higher quantitative completeness but also yielded results that were highly consistent with those derived from LFQ. These findings support the reliability and robustness of the LBQ dataset and underscore the utility of cross‐validation strategies in proteomic studies.

### 3.2 Functional Analysis

#### 3.2.1 Differentially Abundant Proteins

After applying both the *p* value and log_2_ FC thresholds, the number of DAPs identified in the LBQ dataset varied across contrasts: 30 for CTRL, 532 for EE, 1356 for LNG, 1296 for EE + LNG, and 1209 for S‐23 (all vs. DMSO). The observed variation in the number of DAPs across treatment conditions reflects both shared and potentially distinct molecular effects induced by each compound. As expected, the minimal number of DAPs in the CTRL versus DMSO contrast confirms the relative neutrality of the vehicle at the low concentration used here. It supports the exclusion of these proteins from further analysis. Among the treatment groups, LNG induced the most extensive proteomic changes, followed closely by EE + LNG and S‐23, while EE alone showed a comparatively limited number of DAPs. Notably, this lower number in the EE versus DMSO contrast may be influenced by the presence of phenol red in the proprietary cell culture medium. Phenol red is known to exert weak estrogenic activity [32, 33], potentially pre‐activating estrogen‐responsive pathways and thus diminishing the detectable impact of exogenous EE treatment. As such, it is difficult to determine whether the limited response to EE reflects an actual biological difference or is an artifact of estrogenic background signaling.

**FIGURE 4:**
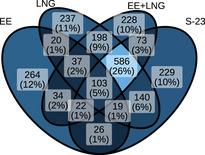
Overlap of DAPs across treatment conditions. Venn diagram showing the overlap of DAPs (based on *p* value and log_2_ FC thresholds) identified in each treatment versus DMSO using the LBQ dataset. The number and percentage of DAPs specific to one or shared between multiple treatments are indicated. Proteins that were differentially abundant in the CTRL versus DMSO contrast were excluded from the dataset.

After the removal of the 30 DAPs from the CTRL versus DMSO contrast, the number of reported DAPs per contrast changed a bit. Figure 4 illustrates all the remaining DAPs and their overlaps between two or several contrasts.

The identification of 103 DAPs shared across all treatment conditions points to a common set of molecular responses to hormonal or hormone‐like stimuli. Additionally, 586 DAPs were found to be common to the LNG, EE + LNG, and S‐23 treatments but not to EE. Although this might suggest overlapping regulatory mechanisms distinct from those influenced by estrogenic pathways, the confounding presence of phenol red precludes firm conclusions. It remains possible that EE would have elicited a broader proteomic response in the absence of phenol red. The presence of treatment‐specific DAPs for each compound also indicates unique regulatory effects; however, cautious interpretation is advised due to the medium‐related limitations.

#### 3.2.2 Enrichment Analyses

The enrichment analyses were performed on the top 200 DAPs per treatment condition (DAP overlap shown in Figure S8) and revealed a diverse array of biological processes and disease associations (Figures S9–S12), with some entries suggesting potential relevance to mental health. However, given that these findings are based on an in vitro system, it is important to interpret the results with caution, recognizing the limitations inherent to the model.

**FIGURE 5:**
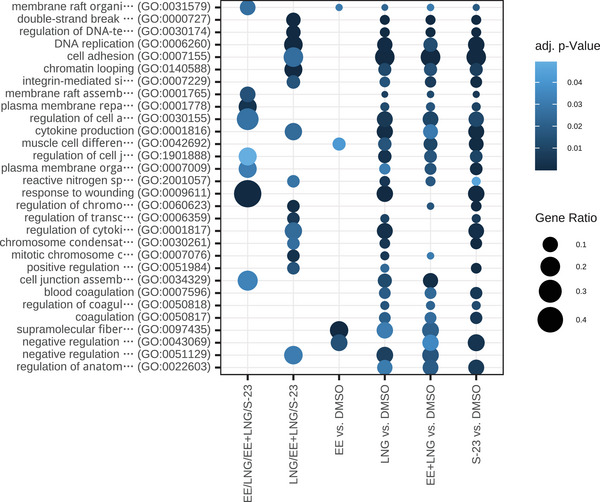
Enriched GO terms. Bubble plot showing the top 30 GO‐enriched biological processes across different experimental contrasts and their intersections. Each row represents a GO term, while columns represent specific pairwise comparisons: EE/LNG/EE + LNG/S‐23, LNG/EE + LNG/S‐23, EE versus DMSO, LNG versus DMSO, EE + LNG versus DMSO, and S‐23 versus DMSO. The size of each bubble reflects the gene ratio, and the color intensity indicates the adjusted *p* value, with darker shades representing stronger statistical significance. This visualization highlights shared, and unique biological processes affected under various treatment conditions.

Among the enriched GO biological processes (see Figure 5), several terms such as cytokine production, reactive nitrogen species metabolism, and regulation of programmed cell death intersect with pathways previously implicated in the pathophysiology of mood disorders [34, 35, 36, 37]. Dysregulated inflammatory signaling, oxidative stress, and altered cell survival pathways have all been associated with depressive phenotypes in both preclinical and clinical studies [38, 39, 40, 41, 42]. Furthermore, the enrichment of processes related to chromatin organization and transcriptional regulation may hint at broader epigenetic or transcriptional reprogramming—mechanisms increasingly linked to stress responses and mental health conditions [43, 44, 45, 46].

KEGG pathway analysis supported these observations to some extent, with enrichment in pathways such as ferroptosis and cytoskeleton regulation. Although these pathways have roles in cell cycle control and tissue architecture, they also play emerging roles in neural function, neuroinflammation, and neurodegeneration [47, 48, 49, 50].

Disease enrichment analyses (DOSE and DisGeNET) yielded numerous associations with neurodegenerative conditions such as Alzheimer's disease and tauopathies, which share mechanistic features with depression, including neuroinflammation [40, 51], synaptic dysfunction [52, 53], and chronic stress [54]. These findings suggest the potential relevance of treatment‐induced proteomic changes to pathways associated with mood disorders.

In addition to processes potentially related to mental health, several enriched disease associations point toward breast cancer, including subtypes such as HER2‐positive and basal‐like breast cancer. This is particularly noteworthy given the established clinical association between long‐term hormonal contraceptive use and an increased risk of breast cancer [55, 56]. Although cancer‐related terms are generally overrepresented in enrichment databases due to their extensive study and annotation [57], the specific appearance of breast cancer subtypes may reflect hormone‐sensitive regulatory mechanisms affected by the treatments.

In contrast to the overrepresented cancer‐related terms, depression, and other psychiatric disorders remain comparatively underrepresented in enrichment databases, reflecting both the complexity of their underlying biology and historical research focus. As a result, the absence of prominent depression‐specific terms should not be taken as evidence against psychiatric relevance. Instead, the presence of mechanistically related processes—such as those tied to oxidative stress, inflammation, and cellular resilience—may indicate indirect but meaningful links to mental health outcomes that warrant further investigation in more disease‐relevant models.

Additionally, the undifferentiated state of the ReNcell VM cells limits their representativeness of mature neuronal or glial populations, potentially skewing the functional interpretation of the results toward general progenitor cell biology or pathways related to proliferation. This effect may be further amplified by the cells’ immortalized nature, which inherently favors proliferative signaling; thus even when compared to matched controls, treatment‐induced modulations may disproportionately engage cancer‐ or growth‐related networks that reflect the cellular context rather than specific disease mechanisms.

## 4 Conclusion

The data and findings from this study suggest that treatment with hormonal compounds commonly found in oral contraceptives—such as EE, LNG, and their combination—as well as the SARM S‐23, can alter protein expression profiles in human neural progenitor cells in vitro. Enrichment analyses revealed changes in biological processes and disease associations with potential relevance to mental health, particularly in pathways implicated in depression. Although these findings may hint at a molecular basis for the observed clinical association between hormonal contraceptive use and increased risk of mood disorders, it is important to interpret the results within the limitations of the experimental model.

This in vitro system represents only a simplified snapshot of the complex physiological effects of hormonal treatments and does not account for key pharmacokinetic factors such as oral absorption, hepatic metabolism, systemic distribution, blood‐brain barrier permeability, or indirect effects mediated through other tissues. Additionally, the undifferentiated state of the ReNcell VM cells does not reflect mature neuronal networks in toto. Thus, while the results raise important hypotheses, they do not establish causality or direct mechanistic links between hormonal exposure and mental illness.

Further studies using more physiologically representative models—such as differentiated neural cultures, organoids, or in vivo systems—are needed to better understand the potential neuropsychiatric consequences of hormonal contraceptive use and related compounds.

## Author Contributions

**Sam Thilmany:** conceptualization, methodology, software, validation, formal analysis, investigation, data curation, writing – original draft, writing – review & editing, visualization, funding acquisition. **Andreas Thomas:** conceptualization, methodology, writing – review & editing, project administration, funding acquisition. **Yvonne Reinders:** methodology, formal analysis, investigation, writing – review & editing. **Farhad Shakeri:** formal analysis, writing – review & editing. **Matthias Vogel:** conceptualization, writing – review & editing, project administration, funding acquisition. **Albert Sickmann:** resources, writing – review & editing, supervision. **Catharina Scholl:** conceptualization, resources, writing – review & editing, supervision, project administration, funding acquisition. **Mario Thevis:** conceptualization, resources, writing – review & editing, supervision, project administration, funding acquisition.

## Conflicts of Interest

The authors have declared no conflict of interest.

## Supporting information




**Supporting File 1:** prca70017‐sup‐0001‐SuppMat.pdf

## Data Availability

The mass spectrometry proteomics data have been deposited to the ProteomeXchange Consortium via the PRIDE [58] partner repository with the dataset identifier PXD056721. The R pipeline used for data analysis and visualization of the raw and processed data is accessible on GitHub at https://github.com/SamThilmany/ILLUMINE‐202401_Analysis‐Pipeline.

## References

[1] B. Asbell , The Pill: A Biography of the Drug That Changed the World (Random House, 1995).

[2] C. Goldin and L. F. Katz , “The Power of the Pill: Oral Contraceptives and Women's Career and Marriage Decisions,” Journal of Political Economy 110, no. 4 (2002): 730–770, 10.1086/340778.

[3] A. Lethaby , M. R. Wise , M. A. J. Weterings , M. Bofill Rodriguez , and J. Brown , “Combined Hormonal Contraceptives for Heavy Menstrual Bleeding,” Cochrane Database of Systematic Reviews , no. 2 (2019), 10.1002/14651858.CD000154.pub3. PMC6369862 30742315

[4] J. Brown , T. J. Crawford , S. Datta , and A. Prentice , “Oral Contraceptives for Pain Associated With Endometriosis,” Cochrane Database of Systematic Reviews , no. 5 (2018), 10.1002/14651858.CD001019.pub3. PMC6494634 29786828

[5] A. O. Arowojolu , M. F. Gallo , L. M. Lopez , D. A. Grimes , and S. E. Garner , “Combined Oral Contraceptive Pills for Treatment of Acne,” Cochrane Database of Systematic Reviews , no. 6 (2012), 10.1002/14651858.CD004425.pub5. 17253506

[6] World Anti‐Doping Agency . 2022 Anti‐Doping Testing Figures. Retrieved 2024‐08‐26 from, https://www.wada‐ama.org/sites/default/files/2024‐04/2022_anti‐doping_testing_figures_en.pdf.

[7] R. P. Dobash , L. Monaghan , R. E. Dobash , and M. Bloor , in Crime Unlimited? Questions for the 21st Century, ed. P. Carlen and R. Morgan (Palgrave Macmillan UK, 1999), 166–190.

[8] A. J. Ferrari , D. F. Santomauro , A. Aali , et al., “Global Incidence, Prevalence, Years Lived With Disability (YLDs), Disability‐Adjusted Life‐Years (DALYs), and Healthy Life Expectancy (HALE) for 371 Diseases and Injuries in 204 Countries and Territories and 811 Subnational Locations, 1990–2021: A Systematic Analysis for the Global Burden of Disease Study 2021,” Lancet 403 (2024): 2133–2161, 10.1016/S0140-6736(24)00757-8. PMC11122111 38642570

[9] P. Greenberg , A. Chitnis , D. Louie , et al., “The Economic Burden of Adults With Major Depressive Disorder in the United States (2019),” Advances in Therapy 40, no. 10 (2023): 4460–4479, 10.1007/s12325-023-02622-x. 37518849 PMC10499687

[10] Institute for Health Metrics and Evaluation (IHME) . GBD Compare Data Visualization. IHME, University of Washington. Retrieved 2024‐09‐11 from http://vizhub.healthdata.org/gbd‐compare.

[11] M. Z. Kraft , P. Rojczyk , T. Weiss , et al., “Symptoms of Mental Disorders and Oral Contraception Use: A Systematic Review and Meta‐Analysis,” Frontiers in Neuroendocrinology 72 (2024): 101111, 10.1016/j.yfrne.2023.101111. 37967755

[12] C. Anderl , A. E. de Wit , E. J. Giltay , A. J. Oldehinkel , and F. S. Chen , “Association Between Adolescent Oral Contraceptive Use and Future Major Depressive Disorder: A Prospective Cohort Study,” Journal of Child Psychology and Psychiatry and Allied Disciplines 63, no. 3 (2022): 333–341, 10.1111/jcpp.13476. 34254301 PMC9291927

[13] C. W. Skovlund , L. S. Morch , L. V. Kessing , and O. Lidegaard , “Association of Hormonal Contraception With Depression,” JAMA Psychiatry 73, no. 11 (2016): 1154–1162, 10.1001/jamapsychiatry.2016.2387. 27680324

[14] S. McKetta and K. M. Keyes , “Oral Contraceptive Use and Depression Among Adolescents,” Annals of Epidemiology 29 (2019): 46–51, 10.1016/j.annepidem.2018.10.002. 30674431 PMC6349422

[15] M. W. L. Morssinkhof , D. W. van Wylick , S. Priester‐Vink , et al., “Associations Between Sex Hormones, Sleep Problems and Depression: A Systematic Review,” Neuroscience and Biobehavioral Reviews 118 (2020): 669–680, 10.1016/j.neubiorev.2020.08.006. 32882313

[16] W. H. Shrank , A. R. Patrick , and M. Alan Brookhart , “Healthy User and Related Biases in Observational Studies of Preventive Interventions: A Primer for Physicians,” Journal of General Internal Medicine 26, no. 5 (2011): 546–550, 10.1007/s11606-010-1609-1. 21203857 PMC3077477

[17] J. A. Kopec and J. M. Esdaile , “Bias in Case‐Control Studies. A Review,” Journal of Epidemiology and Community Health 44, no. 3 (1990): 179, 10.1136/jech.44.3.179. 2273353 PMC1060638

[18] A. Freeman , S. Tyrovolas , A. Koyanagi , et al., “The Role of Socio‐Economic Status in Depression: Results From the COURAGE (Aging Survey in Europe),” BMC Public Health 16, no. 1 (2016): 1098, 10.1186/s12889-016-3638-0. 27760538 PMC5069819

[19] J. Sarris , R. Thomson , F. Hargraves , et al., “Multiple Lifestyle Factors and Depressed Mood: A Cross‐Sectional and Longitudinal Analysis of the UK Biobank (N = 84,860),” BMC Medicine 18, no. 1 (2020): 354, 10.1186/s12916-020-01813-5. 33176802 PMC7661271

[20] J. Y. Song , C. D. Patton , R. Friedman , et al., “Hormonal Contraceptives and the Brain: A Systematic Review on 60 Years of Neuroimaging, EEG, and Biochemical Studies in Humans and Animals,” Frontiers in Neuroendocrinology 68 (2023): 101051, 10.1016/j.yfrne.2022.101051. 36577486 PMC9898167

[21] T. Strowitzki , in Arzneiverordnungs‐Report 2022, ed. W.‐D. Ludwig , B. Mühlbauer and R. Seifert (Springer Berlin Heidelberg, 2022), 733–747.

[22] A. Jones , J. Chen , D. J. Hwang , D. D. Miller , and J. T. Dalton , “Preclinical Characterization of a (S)‐N‐(4‐cyano‐3‐Trifluoromethyl‐Phenyl)‐3‐(3‐Fluoro, 4‐Chlorophenoxy)‐2‐Hydroxy‐2‐Methyl‐Propanamide: A Selective Androgen Receptor Modulator for Hormonal Male Contraception,” Endocrinology 150, no. 1 (2009): 385–395, 10.1210/en.2008-0674. 18772237 PMC2630904

[23] World Anti‐Doping Agency . The 2024 Prohibited List. Retrieved 2024‐08‐01 from, https://www.wada‐ama.org/sites/default/files/2023‐09/2024list_en_final_22_september_2023.pdf.

[24] M. Choi , C. Y. Chang , T. Clough , et al., “MSstats: An R Package for Statistical Analysis of Quantitative Mass Spectrometry‐Based Proteomic Experiments,” Bioinformatics 30, no. 17 (2014): 2524–2526, 10.1093/bioinformatics/btu305. 24794931

[25] T. Huang , M. Choi , M. Tzouros , et al., “MSstatsTMT: Statistical Detection of Differentially Abundant Proteins in Experiments With Isobaric Labeling and Multiple Mixtures,” Molecular & Cellular Proteomics 19, no. 10 (2020): 1706–1723, 10.1074/mcp.RA120.002105. 32680918 PMC8015007

[26] G. Yu , L.‐G. Wang , Y. Han , and Q.‐Y. He , “clusterProfiler: An R Package for Comparing Biological Themes Among Gene Clusters,” OMICS: A Journal of Integrative Biology 16, no. 5 (2012): 284–287, 10.1089/omi.2011.0118. 22455463 PMC3339379

[27] J. Piñero , N. Queralt‐Rosinach , À. Bravo , et al., “DisGeNET: A Discovery Platform for the Dynamical Exploration of Human Diseases and Their Genes,” Database 2015 (2015): bav028, 10.1093/database/bav028. 25877637 PMC4397996

[28] G. Yu , L.‐G. Wang , G.‐R. Yan , and Q.‐Y. He , “DOSE: An R/Bioconductor Package for Disease Ontology Semantic and Enrichment Analysis,” Bioinformatics 31, no. 4 (2015): 608–609, 10.1093/bioinformatics/btu684. 25677125

[29] C. Hutchinson‐Bunch , J. A. Sanford , J. R. Hansen , et al., “Assessment of TMT Labeling Efficiency in Large‐Scale Quantitative Proteomics: The Critical Effect of Sample pH,” ACS Omega 6, no. 19 (2021): 12660–12666, 10.1021/acsomega.1c00776. 34056417 PMC8154127

[30] J. D. O'Connell , J. A. Paulo , J. J. O'Brien , and S. P. Gygi , “Proteome‐Wide Evaluation of Two Common Protein Quantification Methods,” Journal of Proteome Research 17, no. 5 (2018): 1934–1942, 10.1021/acs.jproteome.8b00016. 29635916 PMC5984592

[31] C. Lazar , L. Gatto , M. Ferro , C. Bruley , and T. Burger , “Accounting for the Multiple Natures of Missing Values in Label‐Free Quantitative Proteomics Data Sets to Compare Imputation Strategies,” Journal of Proteome Research 15, no. 4 (2016): 1116–1125, 10.1021/acs.jproteome.5b00981. 26906401

[32] K. G. Rajendran , T. Lopez , and I. Parikh , “Estrogenic Effect of Phenol Red in MCF‐7 Cells Is Achieved Through Activation of Estrogen Receptor by Interacting With a Site Distinct From the Steroid Binding Site,” Biochemical and Biophysical Research Communications 142, no. 3 (1987): 724–731, 10.1016/0006-291X(87)91474-4. 3827898

[33] Y. Berthois , J. A. Katzenellenbogen , and B. S. Katzenellenbogen , “Phenol Red in Tissue Culture media Is a Weak Estrogen: Implications Concerning the Study of Estrogen‐Responsive Cells in Culture,” Proceedings of the National Academy of Sciences 83, no. 8 (1986): 2496–2500, 10.1073/pnas.83.8.2496. PMC323325 3458212

[34] S. Harsanyi , I. Kupcova , L. Danisovic , and M. Klein , “Selected Biomarkers of Depression: What Are the Effects of Cytokines and Inflammation?,” International Journal of Molecular Sciences 24, no. 1 (2023): 36614020, 10.3390/ijms24010578. PMC9820159 36614020

[35] P. Kudlow , D. S. Cha , A. F. Carvalho , and R. S. McIntyre , “Nitric Oxide and Major Depressive Disorder: Pathophysiology and Treatment Implications,” Current Molecular Medicine 16, no. 2 (2016): 206–215, 10.2174/1566524016666160126144722. 26812915

[36] A. George and M. Michael , “Oxidative/Nitrosative Stress and Immuno‐Inflammatory Pathways in Depression: Treatment Implications,” Current Pharmaceutical Design 20, no. 23 (2014): 3812–3847, 10.2174/13816128113196660738. 24180395

[37] S. Li , Y. Sun , M. Song , et al., “NLRP3/Caspase‐1/GSDMD–Mediated Pyroptosis Exerts a Crucial Role in Astrocyte Pathological Injury in Mouse Model of Depression,” JCI Insight 6, no. 23 (2021), 10.1172/jci.insight.146852. PMC8675200 34877938

[38] L. Cui , S. Li , S. Wang , et al., “Major Depressive Disorder: Hypothesis, Mechanism, Prevention and Treatment,” Signal Transduction and Targeted Therapy 9, no. 1 (2024): 30, 10.1038/s41392-024-01738-y. 38331979 PMC10853571

[39] N. Guo , X. Wang , M. Xu , J. Bai , H. Yu , and Z. Le , “PI3K/AKT Signaling Pathway: Molecular Mechanisms and Therapeutic Potential in Depression,” Pharmacological Research 206 (2024): 107300, 10.1016/j.phrs.2024.107300. 38992850

[40] R. Troubat , P. Barone , S. Leman , et al., “Neuroinflammation and Depression: A Review,” European Journal of Neuroscience 53, no. 1 (2021): 151–171, 10.1111/ejn.14720. 32150310

[41] C. D'Sa and R. S. Duman , “Antidepressants and Neuroplasticity,” Bipolar Disorders 4, no. 3 (2002): 183–194, 10.1034/j.1399-5618.2002.01203.x. 12180273

[42] A. N. Tartt , M. B. Mariani , R. Hen , J. J. Mann , and M. Boldrini , “Dysregulation of Adult Hippocampal Neuroplasticity in Major Depression: Pathogenesis and Therapeutic Implications,” Molecular Psychiatry 27, no. 6 (2022): 2689–2699, 10.1038/s41380-022-01520-y. 35354926 PMC9167750

[43] H. Sun , P. J. Kennedy , and E. J. Nestler , “Epigenetics of the Depressed Brain: Role of Histone Acetylation and Methylation,” Neuropsychopharmacology 38, no. 1 (2013): 124–137, 10.1038/npp.2012.73. 22692567 PMC3521990

[44] Z. Duan and J. Lu , “DNA Methyltransferases in Depression: An Update,” Frontiers in Psychiatry 11 (2020): 538683, 10.3389/fpsyt.2020.538683. 1664‐0640 (Print). 33101076 PMC7495306

[45] M. Yuan , B. Yang , G. Rothschild , et al., “Epigenetic Regulation in Major Depression and Other Stress‐Related Disorders: Molecular Mechanisms, Clinical Relevance and Therapeutic Potential,” Signal Transduction and Targeted Therapy 8, no. 1 (2023): 309, 10.1038/s41392-023-01519-z. 37644009 PMC10465587

[46] S. Uchida , H. Yamagata , T. Seki , and Y. Watanabe , “Epigenetic Mechanisms of Major Depression: Targeting Neuronal Plasticity,” Psychiatry and Clinical Neurosciences 72, no. 4 (2018): 212–227, 10.1111/pcn.12621. 29154458

[47] Y. Wang , H. Li , Q. He , R. Zou , J. Cai , and L. Zhang , “Ferroptosis: Underlying Mechanisms and Involvement in Neurodegenerative Diseases,” Apoptosis 29, no. 1 (2024): 3–21, 10.1007/s10495-023-01902-9. 37848673

[48] T. Lan , T. T. Sun , C. Wei , et al., “Epigenetic Regulation of Ferroptosis in Central Nervous System Diseases,” Molecular Neurobiology 60, no. 7 (2023): 3584–3599, 10.1007/s12035-023-03267-1. 36847936

[49] M. L. Green , A. V. Singh , Y. Zhang , K. A. Nemeth , K. K. Sulik , and T. B. Knudsen , “Reprogramming of Genetic Networks During Initiation of the Fetal Alcohol Syndrome,” Developmental Dynamics 236, no. 2 (2007): 613–631, 10.1002/dvdy.21048. 17200951

[50] Q. Bai , J. Liu , and G. Wang , “Ferroptosis, a Regulated Neuronal Cell Death Type After Intracerebral Hemorrhage,” Frontiers in Cellular Neuroscience 14 (2020): 591874, 10.3389/fncel.2020.591874. 33304242 PMC7701249

[51] M. Ly , G. Z. Yu , A. Mian , et al., “Neuroinflammation: A Modifiable Pathway Linking Obesity, Alzheimer's Disease, and Depression,” American Journal of Geriatric Psychiatry 31, no. 10 (2023): 853–866, 10.1016/j.jagp.2023.06.001. PMC10528955 37365110

[52] L. Colucci‐D'Amato , L. Speranza , and F. Volpicelli , “Neurotrophic Factor BDNF, Physiological Functions and Therapeutic Potential in Depression, Neurodegeneration and Brain Cancer,” International Journal of Molecular Sciences 21, (2020): 33096634, 10.3390/ijms21207777. PMC7589016 33096634

[53] S.‐J. Yang , J.‐J. Wang , P. Cheng , L.‐X. Chen , J.‐M. Hu , and G.‐Q. Zhu , “Ginsenoside Rg1 in Neurological Diseases: From Bench to Bedside,” Acta Pharmacologica Sinica 44, no. 5 (2023): 913–930, 10.1038/s41401-022-01022-1. 36380226 PMC10104881

[54] C. Dioli , G. Papadimitriou , A. Megalokonomou , C. Marques , N. Sousa , and I. Sotiropoulos , in GeNeDis 2022, ed. P. Vlamos (Springer International Publishing, 2023), 303–315. 10.1007/978-3-031-31978-5_31 37525058

[55] N. Fakhri , M. A. Chad , M. Lahkim , et al., “Risk Factors for Breast Cancer in Women: An Update Review,” Medical Oncology 39, no. 12 (2022): 197, 10.1007/s12032-022-01804-x. 36071255

[56] D. Fitzpatrick , K. Pirie , G. Reeves , J. Green , and V. Beral , “Combined and Progestagen‐Only Hormonal Contraceptives and Breast Cancer Risk: A UK Nested Case–Control Study and Meta‐Analysis,” PLOS Medicine 20, no. 3 (2023): 1004188, 10.1371/journal.pmed.1004188. PMC10030023 36943819

[57] W. A. Haynes , A. Tomczak , and P. Khatri , “Gene Annotation Bias Impedes Biomedical Research,” Scientific Reports 8, no. 1 (2018): 1362, 10.1038/s41598-018-19333-x. 29358745 PMC5778030

[58] Y. Perez‐Riverol , A. Csordas , J. Bai , et al., “The PRIDE Database and Related Tools and Resources in 2019: Improving Support for Quantification Data,” Nucleic Acids Research 47, no. D1 (2019): D442–D450, 10.1093/nar/gky1106. 30395289 PMC6323896

