# Supplementary material for: Hormonal Contraceptives and Depression: A Proteomic Analysis Using Neuronal Models
Source: Proteomics Clin Appl. 2025 Sep 10;20(1):e70017. doi: 10.1002/prca.70017 (PMC12745671; doi:10.1002/prca.70017)
Supplement: Supplementary file 1 — Supporting File 1: prca70017‐sup‐0001‐SuppMat.pdf [file PRCA-20-e70017-s001.pdf]

Hormonal contraceptives and depression: A proteomic analysis in neuronal models

Supplemental Information

Sam Thilmany[1,2], Andreas Thomas[1], Yvonne Reinders[3], Farhad Shakeri[4], Matthias Vogel[2], Albert Sickmann[3], Catharina Scholl[2], Mario Thevis[1,5]

[1] Institute of Biochemistry, Center for Preventive Doping Research, German Sport University Cologne, Cologne, Germany

[2] Federal Institute for Drugs and Medical Devices, Bonn, Germany

[3] Leibniz-Institut für Analytische Wissenschaften – ISAS – e.V., Dortmund, Germany

[4] Core Unit for Bioinformatics Data Analysis, Medical Faculty, University of Bonn, Bonn, Germany

[5] European Monitoring Center for Emerging Doping Agents (EuMoCEDA), Cologne/Bonn, Germany

List of Figures

Figure S1 Distribution of normalized reporter ion intensities across TMTpro 16plex channels. 4

Figure S2 Evaluation of missing values in LFQ and LBQ datasets. 5

Figure S3 Evaluation of missing values in LFQ and LBQ datasets after filtering. 5

Figure S4 Protein abundance distributions before and after normalization for LFQ and LBQ datasets. 6

Figure S5 Overlap of quantified proteins between LFQ and LBQ datasets. 7

Figure S6 Distribution of statistically significant and non-significant proteins across treatment contrasts. 8

Figure S7 Distribution of log_2 fold changes and thresholding of differentially abundant proteins. 9

Figure S8 Overlap of differentially abundant proteins across treatment conditions. 10

Figure S9 Enriched GO terms. 24

Figure S10 Enriched KEGG terms. 25

Figure S11 Enriched DOSE terms. 26

Figure S12 Enriched DisGeNET entries. 27

List of Tables

Table S1 Consumables with product name, manufacturing company, product number and supplier. 2

Table S2 Equipment with product name, manufacturing company, product number and supplier. 3

Table S3 The differentially abundant proteins used for enrichment analyses. 11

Table S4 Top 30 most significantly enriched GO terms. 24

Table S5 All the enriched KEGG pathways. 25

Table S6 Top 30 most significantly enriched DOSE terms. 26

Table S7 Top 30 most significantly enriched DisGeNET entries. 27

1. Materials

Table S1 Consumables with product name, manufacturing company, product number and supplier.

Product Name	Company	Product Number	Supplier
**17-alpha-Ethynylestradiol**	LKT Laboratories	LKT-E7731.1	Biomol GmbH, Hamburg, Germany
**Cell Culture Flasks, TC-treated, Vented cap**	VWR	734-2311 & 734-2315	VWR International GmbH, Darmstadt, Germany
**Cell Dissociation Buffer, enzyme-free, PBS**	Gibco	13151014	Fisher Scientific GmbH, Schwerte, Germany
**DMSO for molecular biology**	Sigma-Aldrich	D8418-50ML	Merck KGaA, Darmstadt, Germany
**Dulbecco's Phosphate Buffered Saline (DPBS)**	Avantor	392-0434	VWR International GmbH, Darmstadt, Germany
**EasyPep MS Sample Prep Kit**	Thermo Scientific	A40006	Fisher Scientific GmbH, Schwerte, Germany
**EmbryoMax DMEM/F12, with L-Glutamine, without HEPES**	Sigma-Aldrich	DF-042	Merck KGaA, Darmstadt, Germany
**Epidermal Growth Factor Protein, Human recombinant**	Sigma-Aldrich	GF144	Merck KGaA, Darmstadt, Germany
**Fibroblast Growth Factor basic Protein, Human recombinant**	Sigma-Aldrich	GF003	Merck KGaA, Darmstadt, Germany
**Formic acid 0.1 % in Acetonitrile ULC/MS - CC/SFC**	Biosolve	019341	Biosolve BV, Valkenswaard, The Netherlands
**Formic acid 0.1 % in Water ULC/MS - CC/SFC**	Biosolve	232441	Biosolve BV, Valkenswaard, The Netherlands
**Laminin, mouse purified**	Sigma-Aldrich	CC095-M	Merck KGaA, Darmstadt, Germany
**Levonorgestrel**	LKT Laboratories	LKT-L1684.100	Biomol GmbH, Hamburg, Germany
**Pierce Dilution-Free Rapid Gold BCA Protein Assay**	Thermo Scientific	A55861	Fisher Scientific GmbH, Schwerte, Germany
**Pierce High pH Reversed-Phase Peptide Fractionation Kit**	Thermo Scientific	84868	Fisher Scientific GmbH, Schwerte, Germany
**ReNcell NSC Maintenance Media**	Sigma-Aldrich	SCM005	Merck KGaA, Darmstadt, Germany
**ReNcell VM Neural Progenitor Cell Lin**	Sigma-Aldrich	SCC008	Merck KGaA, Darmstadt, Germany
**S-23**	/	/	Synthesized in-house
**TMTpro 16plex Label Reagent Set**	Thermo Scientific	A44521	Fisher Scientific GmbH, Schwerte, Germany
**S-Trap**	ProtiFi	/	ProtiFi, Fairport, NY, US

Table S2 Equipment with product name, manufacturing company, product number and supplier.

Product Name	Company	Product Number	Supplier
**Countess 3 Automatic Cell Counter**	Invitrogen	AMQAX20000	Fisher Scientific GmbH, Schwerte, Germany
**Multiskan FC Microplate Photometer**	Thermo Scientific	1410101	Fisher Scientific GmbH, Schwerte, Germany
**nanoACQUITY UPLC**	Waters	176016000	Waters GmbH, Eschborn, Germany
**NanoDrop One Microvolume UV-Vis Spectrophotometer**	Thermo Scientific	ND-ONE-W	Fisher Scientific GmbH, Schwerte, Germany
**Nanospray Flex Ion Source**	Thermo Scientific	ES071	Thermo Fisher Scientific (Bremen) GmbH, Bremen, Germany
**PepMap Neo Trap Cartridge**	Thermo Scientific	174500	Fisher Scientific GmbH, Schwerte, Germany
**Acclaim PepMap 100 C18 HPLC Column**	Thermo Scientific	164570	Fisher Scientific GmbH, Schwerte, Germany
**Proteome Discoverer Software 3.0**	Thermo Scientific	OPTION-31099	Thermo Fisher Scientific GmbH, Dreieich, Germany
**Q Exactive Hybrid Quadrupole-Orbitrap Mass Spectrometer**	Thermo Scientific	/	Thermo Fisher Scientific (Bremen) GmbH, Bremen, Germany
**Q Exactive HF Hybrid Quadrupole-Orbitrap Mass Spectrometer**	Thermo Scientific	/	Thermo Fisher Scientific (Bremen) GmbH, Bremen, Germany
**Ultimate 3000 Rapid Separation Liquid chromatography**	Thermo Scientific	/	Thermo Fisher Scientific GmbH, Dreieich, Germany
**ReproSil-Pur 120 C18-AQ**	Dr. Maisch	r13.aq.	Dr. A. Maisch HPLC GmbH, Ammerbuch-Entringen, Germany

2. Results

2.1 Technical Analysis

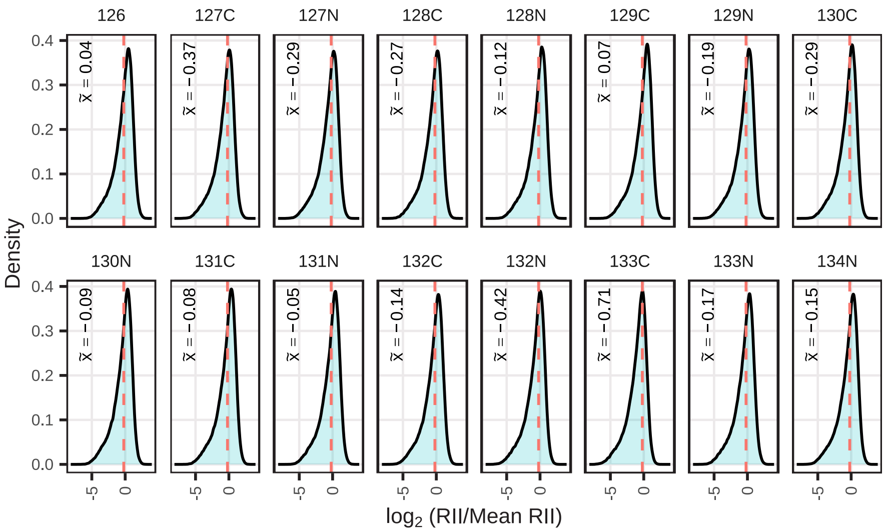

Figure S1 Distribution of normalized reporter ion intensities across TMTpro 16plex channels.
Each density plot represents the distribution of log_2-transformed reporter ion intensities (RII) normalized to the mean RII across all channels (log_2 [RII/Mean RII]) for one of the 16 TMTpro channels. The data is centered approximately around zero (dashed red line) in all channels, indicating consistent labeling efficiency and minimal channel-specific bias.

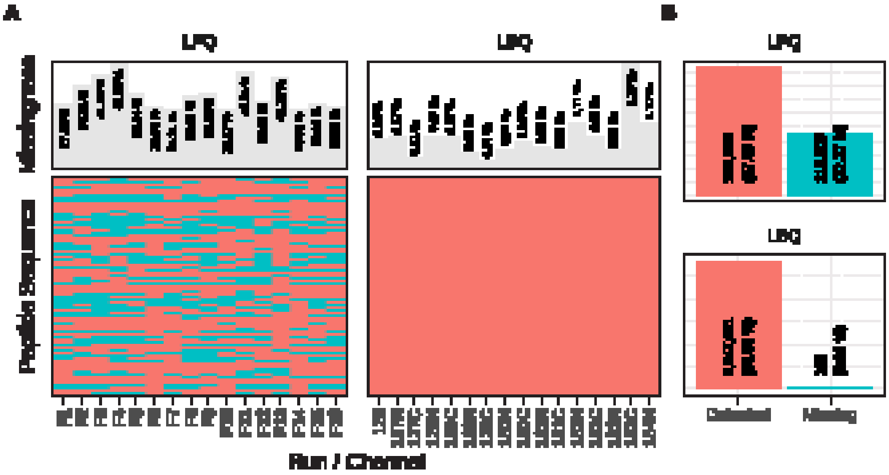

Figure 2 Evaluation of missing values in LFQ and LBQ datasets.
(A) *Missing value distribution per sample (LFQ, left column) and per TMT channel (LBQ, right column). The top panels show the proportion of missing peptide ion intensities per sample or channel as bar plots. The bottom panels display the presence (red) or absence (blue) of intensity values for individual peptide ions across samples or channels, with each row representing a single peptide ion (not aggregated to unique peptides).*
(B) *Overall missingness in the datasets. Bar plots show the proportion of missing intensity values across all peptide ions in the LFQ dataset (top) and the LBQ dataset (bottom).*

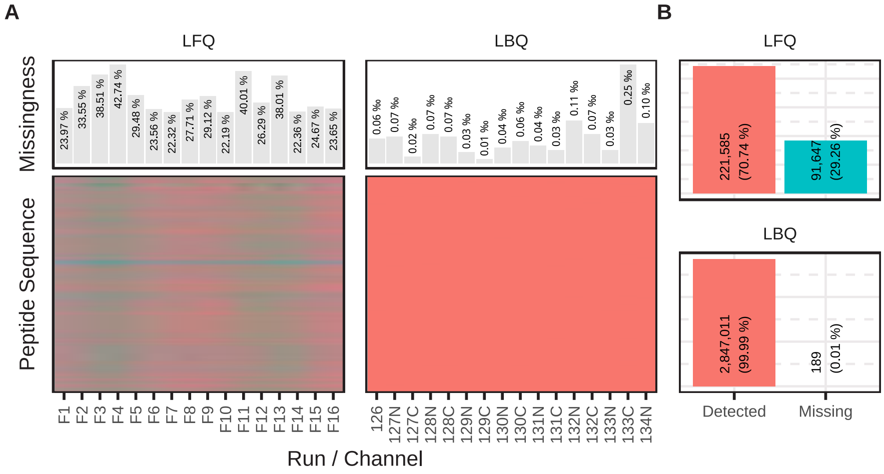

Figure S3 Evaluation of missing values in LFQ and LBQ datasets after filtering.
Only peptide ions with < 50 % missing values across samples (LFQ) or TMT channels (LBQ) were retained prior to visualization.
(A) *Missing value distribution per sample (LFQ, left column) and per TMT channel (LBQ, right column). Top panels show the proportion of missing peptide ion intensities per sample or channel as bar plots. Bottom panels display the presence (red) or absence (blue) of intensity values for individual peptide ions across samples or channels, with each row representing a single peptide ion (not aggregated to unique peptides).*
(B) *Overall missingness in the datasets. Bar plots show the proportion of missing intensity values across all peptide ions in the LFQ dataset (top) and the LBQ dataset (bottom).*

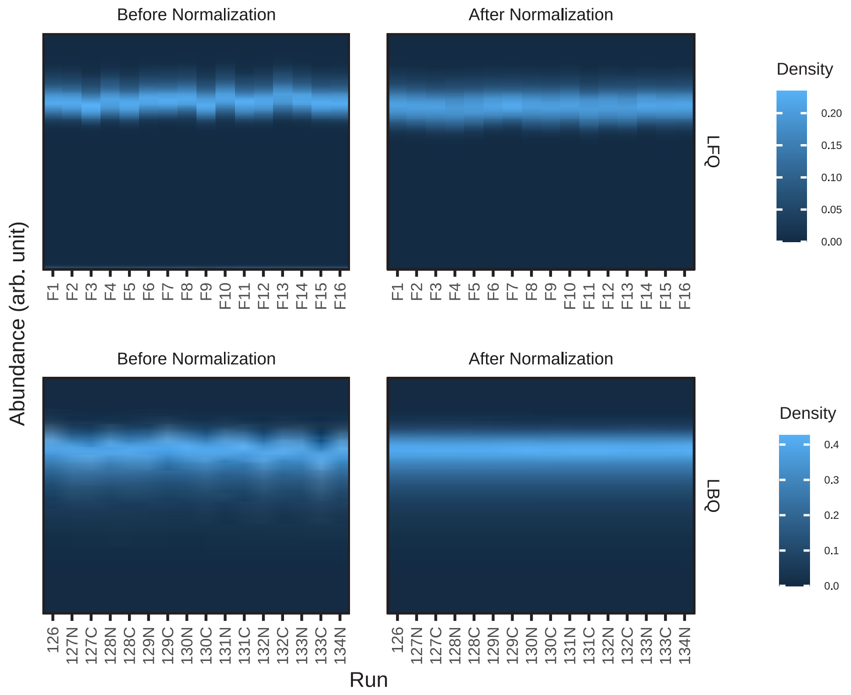

Figure S4 Protein abundance distributions before and after normalization for LFQ and LBQ datasets.
Density heatmaps show the distribution of protein abundances (arbitrary units) across individual LC-HR-MS/MS runs for LFQ data (top row) and across TMT channels for LBQ data (bottom row), before (left) and after (right) protein-level normalization. Prior to normalization, clear systematic differences in abundance levels are observed across both LFQ runs and LBQ channels. After normalization, the abundance distributions are visually aligned, indicating effective correction of inter-sample and inter-channel variation and improved comparability for downstream quantitative analyses.

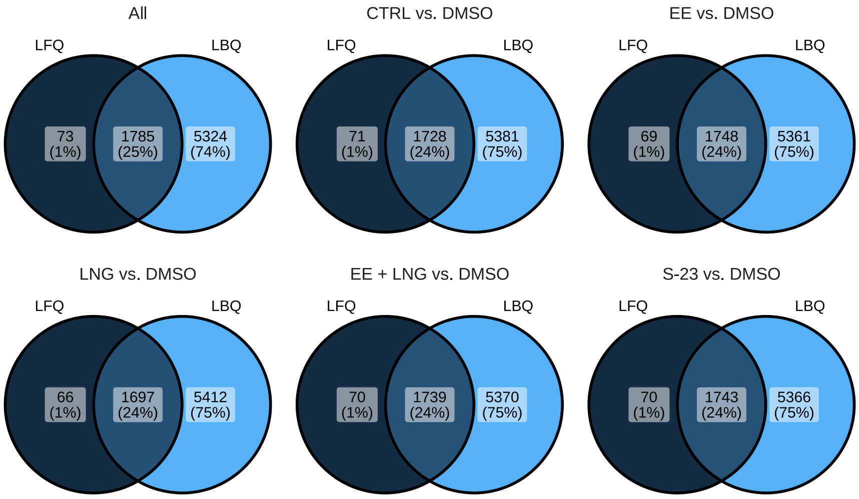

Figure S5 Overlap of quantified proteins between LFQ and LBQ datasets.
Venn diagrams displaying the overlap of proteins quantified by LFQ and LBQ for the full dataset (top left) and for each treatment contrast compared to DMSO (remaining panels). Protein counts are shown prior to applying p-value and log_2 fold change thresholds. The color intensity reflects the number of proteins in each subset, with darker shades indicating smaller sets and lighter shades representing larger ones. Numbers and percentages indicate the absolute and relative proportion of proteins unique to each quantification method or shared between both.

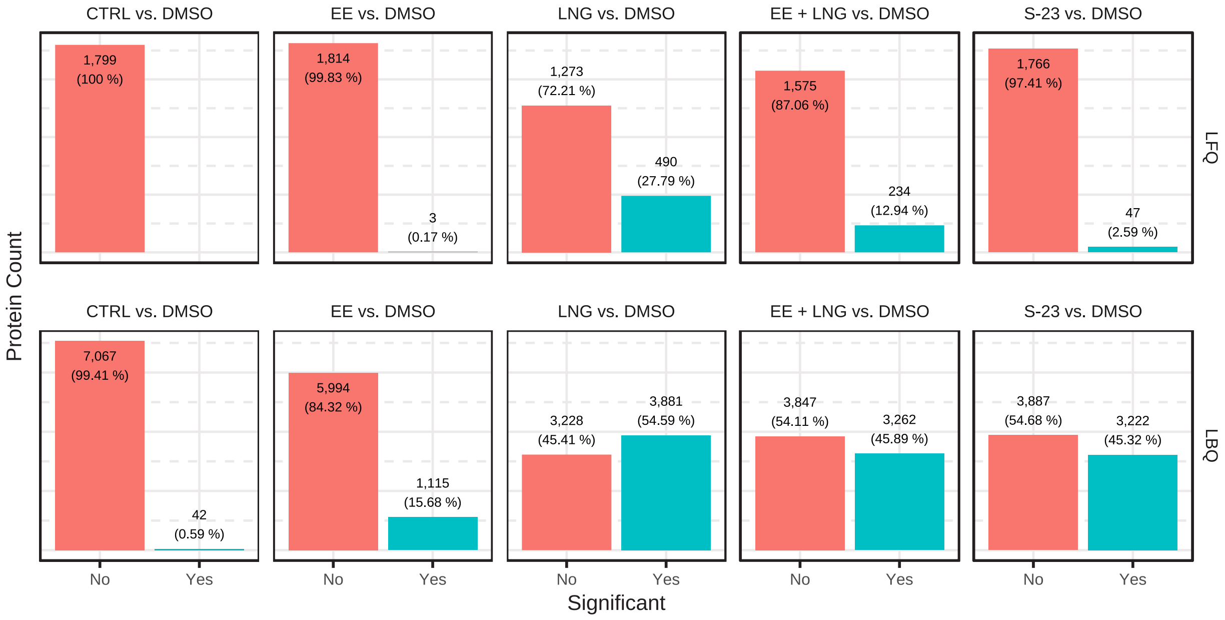

Figure S6 Distribution of statistically significant and non-significant proteins across treatment contrasts.
Bar plots showing the number of proteins quantified in each treatment contrast compared to the DMSO control, for both quantification methods (LFQ, top row; LBQ, bottom row). Within each contrast, proteins are categorized by statistical significance based on adjusted p-values. Proteins with adjusted p-values above the defined false discovery rate (FDR) threshold—0.05 for LFQ and 0.01 for LBQ—are considered non-significant (red), while proteins below the FDR threshold are considered statistically significant (blue). The total number and percentage of proteins in each category are indicated above the respective bars.

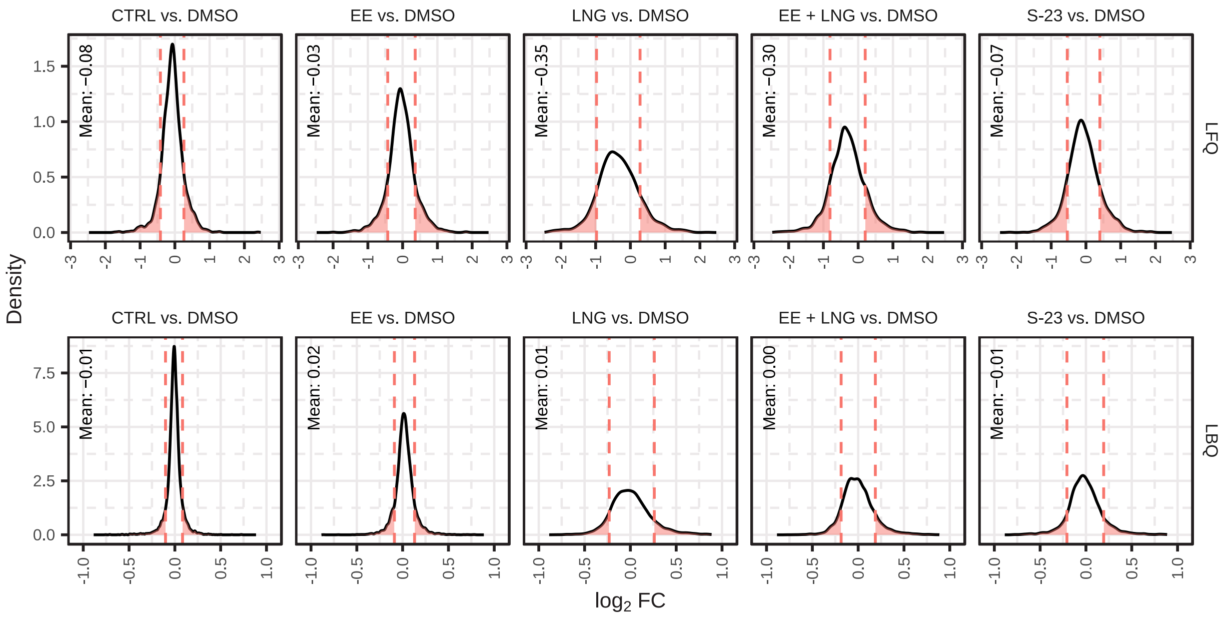

Figure S7 Distribution of log₂ fold changes and thresholding of differentially abundant proteins.

Density plots showing the distribution of $\log_2$ fold change ($\log_2$ FC) values for differentially abundant proteins (DAPs) in each treatment contrast relative to DMSO, for both quantification methods (LFQ, top row; LBQ, bottom row). Red dashed vertical lines indicate the calculated thresholds based on one standard deviation (±SD) from the mean $\log_2$ FC per contrast. Proteins with $\log_2$ FC values outside this range—i.e., greater than (mean + SD) or less than (mean – SD)—are retained and highlighted in red.

2.2 Functional Analysis

Differentially abundant Proteins (DAPs)

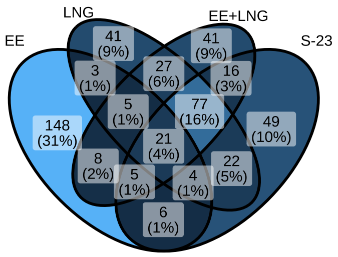

Figure S8 Overlap of differentially abundant proteins across treatment conditions.
Venn diagram showing the overlap of DAPs (based on p-value and $\log_2$ FC thresholds) identified in each treatment vs. DMSO using the LBQ dataset after filtering for the top-200 DAPs per contrast. The number and percentage of DAPs specific to one or shared between multiple treatments are indicated.

Table S3 The differentially abundant proteins used for enrichment analyses.
The top-200 DAPs per contrast were selected. As visualized in Figure S8, this results in 473 unique proteins.

Protein	Gene	Coverage	PSMs	Peptides	Unique Peptides	q-value	XCorr	Treatments
**P52789**	HK2	50	231	38	34	0.000	522.46	EE, EE + LNG, S-23
**Q14195-2**	DPYSL3	69	752	37	31	0.000	1817.85	EE, EE + LNG, LNG, S-23
**P15559**	NQO1	48	316	16	16	0.000	561.64	EE, EE + LNG
**Q99541**	PLIN2	60	151	19	19	0.000	307.34	EE, LNG, S-23
**P07355**	ANXA2	75	1239	30	30	0.000	2568.51	EE, EE + LNG, LNG, S-23
**P40261**	NNMT	51	245	11	11	0.000	578.66	EE, EE + LNG, LNG, S-23
**P21333-2**	FLNA	67	2293	135	117	0.000	5439.96	EE, EE + LNG, LNG, S-23
**P30838**	ALDH3A1	9	18	3	3	0.000	33.92	EE
**P46821**	MAP1B	54	1296	98	96	0.000	2936.34	EE, EE + LNG, LNG, S-23
**Q16643**	DBN1	52	300	25	25	0.000	641.24	EE
**P13797**	PLS3	52	215	29	26	0.000	444.68	EE
**P09972**	ALDOC	58	252	20	13	0.000	697.51	EE
**Q92974**	ARHGEF2	55	270	44	44	0.000	565.49	EE, S-23
**Q12931**	TRAP1	56	505	32	31	0.000	983.11	EE, EE + LNG, LNG
**O00592**	PODXL	23	77	11	11	0.000	168.68	EE
**P00338**	LDHA	68	792	20	18	0.000	1607.65	EE
**P60174**	TPI1	88	625	20	20	0.000	1654.06	EE
**P04083**	ANXA1	67	638	22	22	0.000	1396.63	EE, EE + LNG, LNG, S-23
**Q9Y4K1**		27	149	32	32	0.000	314.75	EE
**Q53FA7**	TP53I3	42	99	12	12	0.000	213.77	EE, EE + LNG, LNG, S-23
**Q14571**	ITPR2	11	115	24	24	0.000	192.53	EE
**Q5T9L3**	WLS	21	71	11	11	0.000	133.72	EE
**P30837**	ALDH1B1	32	67	14	12	0.000	161.80	EE
**P13693**	TPT1	58	221	11	11	0.000	432.76	EE
**P35052**	GPC1	46	175	21	21	0.000	389.46	EE
**P78559**	MAP1A	30	372	67	65	0.000	669.23	EE
**P16070**	CD44	13	204	10	10	0.000	373.97	EE, EE + LNG, LNG, S-23
**P48960**	ADGRE5	8	58	5	5	0.000	124.06	EE
**P31040**	SDHA	54	183	24	24	0.000	437.02	EE, EE + LNG
**P19022**	CDH2	30	161	21	21	0.000	280.66	EE, EE + LNG, S-23
**P55145**	MANF	36	68	6	6	0.000	168.01	EE
**P11166**	SLC2A1	10	27	5	5	0.000	46.66	EE
**P15311**	EZR	66	850	49	32	0.000	1505.36	EE, EE + LNG, LNG, S-23
**P18206**	VCL	72	1209	79	79	0.000	2307.26	EE, EE + LNG, LNG, S-23
**P27144**	AK4	31	30	6	6	0.000	57.06	EE
**P02794**	FTH1	55	41	7	7	0.000	107.10	EE

Protein	Gene	Coverage	PSMs	Peptides	Unique Peptides	q-value	XCorr	Treatments
**Q10471**	GALNT2	51	187	26	25	0.000	349.82	EE
**O75521**	ECI2	46	78	13	13	0.000	181.47	EE
**P02786**	TFRC	47	320	30	30	0.000	591.64	EE
**Q01581**	HMGCS1	64	392	25	25	0.000	974.76	EE
**Q9BVP2**	GNL3	42	155	20	18	0.000	261.65	EE
**P35232**	PHB1	88	357	19	19	0.000	769.68	EE
**Q9HAV7**	GRPEL1	49	115	11	11	0.000	204.63	EE, EE + LNG, LNG
**P09471-2**	GNAO1	29	92	9	4	0.000	191.03	EE
**P50454**	SERPINH1	64	458	22	22	0.000	956.89	EE
**P55769**	SNU13	52	73	6	6	0.000	149.42	EE
**P48507**	GCLM	42	50	9	9	0.000	107.09	EE
**P30536**	TSPO	9	30	2	2	0.000	40.81	EE
**Q16831**	UPP1	28	29	8	8	0.000	51.31	EE
**P36404**	ARL2	55	68	8	8	0.000	108.45	EE
**P02787**	TF	57	331	36	36	0.000	717.42	EE
**P43304**	GPD2	55	213	33	33	0.000	354.93	EE
**P46940**	IQGAP1	61	1047	86	79	0.000	2365.52	EE, EE + LNG
**P20020-1**	ATP2B1	28	151	28	19	0.000	327.49	EE
**P00918**	CA2	56	124	10	10	0.000	230.14	EE
**P04792**	HSPB1	51	273	11	11	0.000	449.86	EE, S-23
**Q02218**	OGDH	39	223	32	32	0.000	410.31	EE
**O95677**	EYA4	15	34	7	7	0.000	74.66	EE
**P48681**	NES	60	1829	89	89	0.000	3401.82	EE
**P23378**	GLDC	49	248	37	37	0.000	569.18	EE
**Q8IZV5**	RDH10	53	51	12	12	0.000	139.28	EE
**Q8N183**	NDUFAF2	41	59	6	6	0.000	83.77	EE
**Q15121**	PEA15	48	109	5	5	0.000	273.18	EE, EE + LNG, LNG, S-23
**Q9UBH6**	XPR1	21	54	11	11	0.000	116.36	EE, LNG
**P01903**	HLA-DRA	11	6	2	2	0.000	15.22	EE
**P08758**	ANXA5	84	781	28	27	0.000	1327.77	EE, EE + LNG, LNG
**P35221**	CTNNA1	61	661	46	45	0.000	1530.39	EE, EE + LNG, LNG, S-23
**Q9NR19**	ACSS2	14	33	9	9	0.000	41.76	EE
**O75056**	SDC3	2	10	1	1	0.000	16.92	EE
**Q53EL6**	PDCD4	45	137	16	16	0.000	303.21	EE
**O14530**	TXNDC9	45	71	10	10	0.000	131.01	EE
**Q14257**	RCN2	62	253	17	17	0.000	532.93	EE
**Q9BQI0**	AIF1L	29	18	3	3	0.000	27.61	EE
**P49821**	NDUFV1	59	137	21	21	0.000	282.50	EE

Protein	Gene	Coverage	PSMs	Peptides	Unique Peptides	q-value	XCorr	Treatments
**Q9NZM1**	MYOF	36	304	63	63	0.000	560.24	EE, EE + LNG, LNG, S-23
**Q9UQB8**	BAIAP2	42	114	21	21	0.000	163.32	EE, S-23
**P32322**	PYCR1	44	55	9	8	0.000	158.76	EE
**O75600**	GCAT	20	32	6	6	0.000	75.52	EE
**Q9Y696**	CLIC4	75	233	16	16	0.000	517.16	EE, EE + LNG
**P35222**	CTNNB1	56	378	36	30	0.000	786.21	EE, LNG, S-23
**Q92686**	NRGN	19	2	1	1	0.024	3.03	EE
**Q14315**	FLNC	42	566	90	72	0.000	1011.32	EE, EE + LNG
**Q9Y3D9**	MRPS23	46	46	7	7	0.000	77.87	EE
**Q9BXW7**	HDHD5	33	67	9	9	0.000	146.53	EE
**Q03154**	ACY1	49	50	14	14	0.000	108.21	EE
**O94760**	DDAH1	59	70	13	12	0.000	138.61	EE
**P34949**	MPI	33	53	11	11	0.000	142.28	EE
**O43676**	NDUFB3	44	47	5	5	0.000	62.52	EE
**Q16718**	NDUFA5	66	81	7	7	0.000	234.80	EE
**Q99653**	CHP1	33	16	5	5	0.000	35.72	EE
**Q7Z4S6**	KIF21A	18	125	23	20	0.000	239.45	EE
**Q00059**	TFAM	38	74	12	12	0.000	96.71	EE, EE + LNG
**P02792**	FTL	28	14	3	3	0.000	38.55	EE
**P23471**	PTPRZ1	22	237	37	37	0.000	451.47	EE
**Q8IV08**	PLD3	41	146	15	15	0.000	324.71	EE, EE + LNG, LNG, S-23
**P0DJ07**	PET100	11	6	1	1	0.001	9.14	EE
**Q9Y2Q9**	MRPS28	51	45	8	8	0.000	99.34	EE
**P50995**	ANXA11	33	83	16	16	0.000	163.13	EE
**Q53EL9**	SEZ6	3	8	2	2	0.000	10.38	EE
**Q9BQ69**	MACROD1	23	19	4	4	0.000	84.66	EE
**Q9UL15**	BAG5	40	80	12	12	0.000	180.00	EE
**P51784**	USP11	23	93	18	16	0.000	176.09	EE
**Q13509**	TUBB3	66	1264	25	8	0.000	2839.55	EE
**P27816**	MAP4	55	740	54	54	0.000	1219.63	EE
**P55327-3**	TPD52	23	66	5	5	0.000	132.07	EE
**Q9Y3D6**	FIS1	36	55	5	5	0.000	103.29	EE
**Q96A26**	FAM162A	33	43	6	6	0.000	88.31	EE
**P00403**	MT-CO2	26	108	5	5	0.000	222.52	EE, EE + LNG
**Q01469**	FABP5	71	215	10	10	0.000	586.82	EE, EE + LNG, LNG, S-23
**P47985**	UQCRFS1	49	195	12	12	0.000	384.86	EE
**Q9UBP4**	DKK3	3	11	1	1	0.000	15.79	EE, S-23
**O95671**	ASMTL	28	59	14	14	0.000	153.95	EE

Protein	Gene	Coverage	PSMs	Peptides	Unique Peptides	q-value	XCorr	Treatments
**Q00534**	CDK6	46	73	11	10	0.000	136.38	EE
**Q8N335**	GPD1L	50	57	16	16	0.000	119.03	EE
**P00813**	ADA	8	19	3	3	0.000	23.34	EE
**Q01518**	CAP1	56	476	25	24	0.000	883.98	EE, EE + LNG, LNG, S-23
**Q15417**	CNN3	60	285	17	16	0.000	564.20	EE, EE + LNG
**Q9BXR0**	QTRT1	23	27	7	7	0.000	53.69	EE
**Q16555**	DPYSL2	76	950	31	26	0.000	2435.07	EE, LNG, S-23
**Q13907**	IDI1	58	112	10	10	0.000	262.44	EE
**P02768**	ALB	74	686	44	44	0.000	2049.46	EE
**Q6IQ22**	RAB12	40	57	9	8	0.000	84.54	EE
**P21912**	SDHB	48	68	12	12	0.000	119.60	EE
**Q02952**	AKAP12	27	147	31	31	0.000	284.43	EE
**O00116**	AGPS	43	120	19	19	0.000	303.73	EE
**O15075**	DCLK1	31	146	20	19	0.000	282.77	EE
**P48163**	ME1	51	300	21	21	0.000	589.50	EE, EE + LNG, LNG
**Q6PI78**	TMEM65	9	8	2	2	0.001	14.40	EE
**Q6YHK3**	CD109	28	159	28	28	0.000	312.01	EE, S-23
**Q658P3**	STEAP3	9	15	3	3	0.000	27.58	EE
**Q13243**	SRSF5	30	93	8	7	0.000	127.71	EE
**P28482**	MAPK1	75	255	22	16	0.000	547.38	EE
**P56559**	ARL4C	31	16	4	4	0.000	41.50	EE
**P21589**	NT5E	47	144	19	19	0.000	305.65	EE
**P60903**	S100A10	36	84	3	3	0.000	116.17	EE, EE + LNG, LNG, S-23
**O00625**	PIR	56	105	12	12	0.000	199.69	EE
**P62304**	SNRPE	52	78	3	3	0.000	162.86	EE
**P34896**	SHMT1	35	67	13	12	0.000	129.45	EE
**Q9NSC5**	HOMER3	30	32	8	8	0.000	66.98	EE
**P11142**	HSPA8	67	1742	39	25	0.000	3547.05	EE
**Q9NUL7**	DDX28	10	12	3	3	0.000	23.51	EE
**Q13576**	IQGAP2	25	168	33	29	0.000	257.39	EE
**Q9UBG0**	MRC2	33	166	34	34	0.000	416.21	EE
**P12236**	SLC25A6	61	501	18	2	0.000	864.93	EE
**P21291**	CSRP1	64	87	10	10	0.000	204.49	EE, LNG
**P06756**	ITGAV	50	327	41	41	0.000	619.97	EE, EE + LNG, LNG, S-23
**O43347**	MSI1	41	165	12	10	0.000	345.02	EE
**P07900**	HSP90AA1	60	2228	44	27	0.000	4385.72	EE, LNG
**Q8NEP3**	DNAAF1	1	12	1	1	0.013	10.66	EE
**Q9BYC8**	MRPL32	12	5	2	2	0.000	12.21	EE

Protein	Gene	Coverage	PSMs	Peptides	Unique Peptides	q-value	XCorr	Treatments
**P52907**	CAPZA1	64	158	11	9	0.000	423.67	EE
**P11047**	LAMC1	20	122	26	26	0.000	215.57	EE
**Q8IWQ3**	BRSK2	2	6	1	1	0.009	6.68	EE
**Q9UBQ7**	GRHPR	59	77	13	13	0.000	211.50	EE, LNG, S-23
**O15540**	FABP7	72	616	8	8	0.000	1277.50	EE, EE + LNG, LNG, S-23
**P78310**	CXADR	27	32	8	8	0.000	66.75	EE
**O43852**	CALU	67	376	19	5	0.000	726.36	EE
**Q9Y2W6**	TDRKH	8	7	3	3	0.000	15.22	EE
**O43818**	RRP9	45	73	16	16	0.000	212.43	EE
**Q9NRG1**	PRTFDC1	32	49	7	6	0.000	85.83	EE
**Q8N257**	H2BC26	79	1150	12	1	0.000	1885.19	EE
**Q9UHB6**	LIMA1	45	171	27	27	0.000	311.66	EE
**O75695**	RP2	30	54	10	10	0.000	98.05	EE
**A4D1E9**	GTPBP10	21	36	8	8	0.000	54.71	EE
**Q9C0D9**	SELENOI	9	10	2	2	0.000	30.85	EE
**Q9Y3C1**	NOP16	54	41	9	9	0.000	76.95	EE
**Q8WUY1**	THEM6	17	21	3	3	0.000	44.68	EE
**Q6NUK4**	REEP3	28	23	6	6	0.000	40.73	EE
**Q96CX2**	KCTD12	11	14	3	3	0.000	20.20	EE
**P38159**	RBMX	59	539	27	10	0.000	676.61	EE
**Q14764**	MVP	42	272	30	30	0.000	518.35	EE, EE + LNG, LNG, S-23
**Q8IX04**	UEVLD	20	32	7	7	0.000	47.82	EE
**P29034**	S100A2	24	9	3	3	0.000	9.31	EE
**P13929**	ENO3	27	393	9	4	0.000	1295.53	EE
**O00469**	PLOD2	23	60	13	13	0.000	106.31	EE
**P26012**	ITGB8	42	111	23	23	0.000	298.12	EE, EE + LNG, S-23
**P24530**	EDNRB	17	24	8	8	0.000	44.17	EE
**Q16527**	CSRP2	61	122	9	9	0.000	287.16	EE
**Q96DG6**	CMBL	46	99	12	12	0.000	165.64	EE
**Q13425**	SNTB2	16	25	8	8	0.000	41.94	EE
**P05166**	PCCB	20	44	8	8	0.000	106.59	EE
**Q6UW68**	TMEM205	21	18	3	3	0.000	37.12	EE
**Q9BUP0**	EFHD1	17	47	4	2	0.000	68.63	EE
**Q9Y6R0**	NUMBL	18	42	11	8	0.000	70.84	EE
**P60842**	EIF4A1	61	830	29	15	0.000	1697.12	EE
**P12110**	COL6A2	19	107	18	18	0.000	197.20	EE, EE + LNG, S-23
**Q27J81**	INF2	25	124	22	21	0.000	311.82	EE, S-23
**P06396**	GSN	42	307	29	29	0.000	611.78	EE

Protein	Gene	Coverage	PSMs	Peptides	Unique Peptides	q-value	XCorr	Treatments
**P12814**	ACTN1	71	1026	56	28	0.000	2312.38	EE, EE + LNG, LNG
**P11413**	G6PD	76	409	34	34	0.000	885.25	EE, EE + LNG, S-23
**Q8N0X4**	CLYBL	13	12	3	3	0.000	21.73	EE
**P54826**	GAS1	2	3	1	1	0.035	2.13	EE
**P10253**	GAA	23	106	15	15	0.000	219.92	EE
**P30049**	ATP5F1D	14	30	2	2	0.000	49.04	EE
**Q9NX74**	DUS2	9	12	3	3	0.000	23.50	EE
**O75880**	SCO1	16	14	4	4	0.000	17.61	EE
**P30622**	CLIP1	17	74	21	16	0.000	127.99	EE
**P53611**	RABGGTB	35	62	9	9	0.000	97.17	EE
**O15498**	YKT6	72	133	12	12	0.000	260.06	EE
**Q8IVF2**	AHNAK2	28	318	63	61	0.000	462.26	EE, EE + LNG, LNG, S-23
**Q9Y490**	TLN1	64	1426	134	116	0.000	2966.72	EE + LNG, LNG, S-23
**P35580**	MYH10	61	1609	124	95	0.000	3454.56	LNG, S-23
**Q13938**	CAPS	25	39	6	6	0.000	83.66	EE + LNG, LNG, S-23
**Q14847**	LASP1	49	109	13	13	0.000	161.26	EE + LNG, LNG, S-23
**P05091**	ALDH2	41	78	16	14	0.000	172.76	EE + LNG, LNG
**P29590**	PML	34	157	28	28	0.000	262.93	EE + LNG, LNG, S-23
**P35579**	MYH9	67	2872	141	114	0.000	6444.76	EE + LNG, LNG, S-23
**P25205**	MCM3	64	463	47	47	0.000	870.25	EE + LNG, LNG, S-23
**Q96B97**	SH3KBP1	24	72	12	12	0.000	151.16	LNG
**Q9UIG0**	BAZ1B	39	313	54	54	0.000	555.12	EE + LNG, LNG, S-23
**Q01082**	SPTBN1	68	1394	132	120	0.000	2896.62	EE + LNG, LNG, S-23
**P34897**	SHMT2	65	431	28	27	0.000	854.07	LNG
**P33992**	MCM5	63	368	39	39	0.000	716.83	EE + LNG, LNG, S-23
**O60664-2**	PLIN3	79	273	15	2	0.000	585.92	EE + LNG, LNG, S-23
**Q08722**	CD47	11	56	4	4	0.000	78.78	EE + LNG, LNG, S-23
**O60664-3**	PLIN3	76	553	25	12	0.000	1175.84	EE + LNG, LNG, S-23
**P35241**	RDX	64	806	48	28	0.000	1572.22	EE + LNG, LNG, S-23
**A1X283**	SH3PXD2B	50	295	36	36	0.000	583.18	EE + LNG, LNG, S-23
**P61916**	NPC2	60	40	7	7	0.000	95.89	EE + LNG, LNG, S-23
**P10809**	HSPD1	72	1488	38	38	0.000	3427.85	EE + LNG, LNG
**Q92747**	ARPC1A	58	107	16	15	0.000	226.36	EE + LNG, LNG
**P07339**	CTSD	45	232	15	15	0.000	547.31	EE + LNG, LNG, S-23
**P49321**	NASP	64	637	40	40	0.000	1468.46	EE + LNG, LNG, S-23
**P29323**	EPHB2	29	123	21	20	0.000	282.56	EE + LNG, LNG, S-23
**Q13451**	FKBP5	66	256	28	28	0.000	515.86	EE + LNG, LNG, S-23
**P11216**	PYGB	67	699	53	43	0.000	1371.94	EE + LNG, LNG, S-23

Protein	Gene	Coverage	PSMs	Peptides	Unique Peptides	q-value	XCorr	Treatments
**Q56VL3**	OCIAD2	51	50	8	8	0.000	101.75	EE + LNG, LNG
**P33991**	MCM4	64	483	47	46	0.000	1063.61	EE + LNG, LNG, S-23
**Q16851**	UGP2	55	185	24	24	0.000	364.91	EE + LNG, LNG
**O94875**	SORBS2	16	63	15	14	0.000	96.36	EE + LNG, LNG, S-23
**P04271**	S100B	33	65	2	2	0.000	171.61	LNG
**Q14566**	MCM6	55	385	43	43	0.000	742.95	EE + LNG, LNG, S-23
**Q08945**	SSRP1	47	269	29	29	0.000	538.02	EE + LNG, LNG, S-23
**Q9NVI1**	FANCI	26	125	24	24	0.000	249.17	EE + LNG, LNG, S-23
**Q9NZJ4**	SACS	32	654	119	119	0.000	1245.12	EE + LNG, LNG, S-23
**Q03135**	CAV1	57	167	10	10	0.000	292.62	EE + LNG, LNG, S-23
**Q6NZI2**	CAVIN1	36	122	13	13	0.000	217.89	EE + LNG, LNG, S-23
**Q9NVP1**	DDX18	52	253	28	28	0.000	482.79	EE + LNG, LNG, S-23
**P09936**	UCHL1	62	316	11	11	0.000	955.55	EE + LNG, LNG, S-23
**P30044**	PRDX5	55	147	10	10	0.000	322.58	EE + LNG, LNG, S-23
**P33993**	MCM7	60	459	36	36	0.000	913.59	EE + LNG, LNG, S-23
**O75368**	SH3BGRL	61	37	6	6	0.000	67.25	LNG
**P04179**	SOD2	56	128	12	12	0.000	234.41	LNG, S-23
**Q9NZ08**	ERAP1	37	137	26	26	0.000	288.37	LNG
**P27658**	COL8A1	6	18	3	3	0.000	33.30	EE + LNG, LNG, S-23
**P11137**	MAP2	35	317	48	32	0.000	601.83	EE + LNG, LNG, S-23
**O95347**	SMC2	44	370	49	49	0.000	665.88	EE + LNG, LNG, S-23
**Q9NQC3-2**	RTN4	34	285	10	1	0.000	572.36	EE + LNG, LNG, S-23
**Q9NR30**	DDX21	58	466	40	38	0.000	944.41	EE + LNG, LNG, S-23
**O95782**	AP2A1	60	361	47	34	0.000	788.20	EE + LNG, LNG, S-23
**Q9H0S4**	DDX47	38	91	13	13	0.000	184.59	EE + LNG, LNG, S-23
**P09486**	SPARC	38	89	10	10	0.000	271.00	EE + LNG, LNG, S-23
**P16144-3**	ITGB4	27	213	36	36	0.000	402.91	EE + LNG, LNG, S-23
**P50993**	ATP1A2	35	289	31	15	0.000	611.32	LNG, S-23
**P17655**	CAPN2	55	318	29	29	0.000	760.07	EE + LNG, LNG, S-23
**O75717**	WDHD1	15	65	13	13	0.000	139.25	EE + LNG, LNG, S-23
**Q14699**	RFTN1	48	131	20	20	0.000	260.48	LNG, S-23
**Q96HC4**	PDLIM5	40	136	19	19	0.000	269.23	EE + LNG, LNG, S-23
**P04632**	CAPNS1	58	192	11	11	0.000	481.52	EE + LNG, LNG, S-23
**P10909**	CLU	31	108	12	12	0.000	204.65	EE + LNG, LNG, S-23
**P11387**	TOP1	49	332	38	37	0.000	568.07	LNG, S-23
**P07384**	CAPN1	41	135	26	26	0.000	267.21	EE + LNG, LNG, S-23
**Q9P258**	RCC2	63	313	27	27	0.000	754.83	EE + LNG, LNG, S-23
**Q09666**	AHNAK	61	1498	188	185	0.000	2269.68	EE + LNG, LNG, S-23

Protein	Gene	Coverage	PSMs	Peptides	Unique Peptides	q-value	XCorr	Treatments
**O75396**	SEC22B	56	148	10	10	0.000	304.45	EE + LNG, LNG
**Q8TEQ6**	GEMIN5	45	280	51	51	0.000	562.52	EE + LNG, LNG, S-23
**Q99986**	VRK1	52	136	16	16	0.000	257.59	LNG, S-23
**Q13510**	ASAH1	46	84	13	13	0.000	138.47	LNG
**P23921**	RRM1	63	481	39	39	0.000	1042.44	EE + LNG, LNG, S-23
**Q9UN36**	NDRG2	26	32	7	7	0.000	61.18	EE + LNG, LNG, S-23
**P49915**	GMPS	67	342	38	38	0.000	719.23	EE + LNG, LNG, S-23
**Q96AQ6**	PBXIP1	27	123	17	17	0.000	209.94	LNG, S-23
**P49368**	CCT3	66	635	35	34	0.000	1360.85	LNG
**P18084**	ITGB5	12	25	8	8	0.000	50.86	EE + LNG, LNG, S-23
**A6NCE7**	MAP1LC3B2	22	45	3	3	0.000	61.19	EE + LNG, LNG
**Q13085**	ACACA	48	582	87	87	0.000	1173.58	EE + LNG, LNG
**Q9Y5B9**	SUPT16H	58	624	54	54	0.000	1190.46	EE + LNG, LNG, S-23
**Q96AY3**	FKBP10	59	318	27	27	0.000	644.30	EE + LNG, LNG
**Q92598**	HSPH1	66	562	45	41	0.000	1271.84	LNG, S-23
**Q9UNQ2**	DIMT1	50	65	12	12	0.000	131.76	EE + LNG, LNG, S-23
**Q9BRX8**	PRXL2A	38	115	9	9	0.000	183.37	LNG, S-23
**Q6NUM9**	RETSAT	29	76	15	15	0.000	137.02	LNG, S-23
**Q9BSJ8**	ESYT1	39	253	34	34	0.000	492.05	LNG
**P08133**	ANXA6	70	730	46	46	0.000	1367.61	LNG
**Q14839**	CHD4	46	565	79	79	0.000	1114.64	EE + LNG, LNG, S-23
**P09543**	CNP	71	276	28	27	0.000	553.70	LNG
**Q06323**	PSME1	66	252	18	18	0.000	445.22	EE + LNG, LNG
**P05026**	ATP1B1	43	110	11	11	0.000	215.63	EE + LNG, LNG, S-23
**Q9NTJ3**	SMC4	49	340	53	53	0.000	617.80	EE + LNG, LNG, S-23
**P55010**	EIF5	55	175	21	21	0.000	327.70	EE + LNG, LNG
**Q9Y606**	PUS1	36	88	13	13	0.000	146.54	EE + LNG, LNG
**P49736**	MCM2	52	368	39	39	0.000	683.68	EE + LNG, LNG, S-23
**O76054**	SEC14L2	45	111	16	13	0.000	212.28	EE + LNG, LNG, S-23
**P51572**	BCAP31	50	175	14	14	0.000	295.16	LNG
**P26358**	DNMT1	30	204	39	39	0.000	364.90	LNG, S-23
**P17676**	CEBPB	8	11	3	3	0.000	15.13	LNG
**O95831**	AIFM1	48	169	22	22	0.000	371.27	EE + LNG, LNG
**Q9UNX4**	WDR3	47	227	35	35	0.000	484.18	EE + LNG, LNG, S-23
**Q9UJ70**	NAGK	45	79	12	12	0.000	149.85	EE + LNG, LNG
**P62330**	ARF6	66	67	7	7	0.000	151.98	EE + LNG, LNG
**Q05682-5**	CALD1	51	224	30	30	0.000	409.34	EE + LNG, LNG
**Q12965**	MYO1E	17	81	17	16	0.000	129.75	EE + LNG, LNG

Protein	Gene	Coverage	PSMs	Peptides	Unique Peptides	q-value	XCorr	Treatments
**Q02790**	FKBP4	66	417	29	29	0.000	771.19	EE + LNG, LNG
**Q14194**	CRMP1	25	104	12	7	0.000	182.35	LNG
**Q15811**	ITSN1	12	53	17	17	0.000	74.63	LNG
**P78316**	NOP14	27	89	20	20	0.000	173.94	EE + LNG, LNG, S-23
**O60341**	KDM1A	47	187	33	33	0.000	394.65	EE + LNG, LNG, S-23
**Q92979**	EMG1	57	114	11	11	0.000	221.97	EE + LNG, LNG
**P40763**	STAT3	52	229	29	29	0.000	530.44	LNG
**Q15021**	NCAPD2	44	288	45	45	0.000	547.46	EE + LNG, LNG, S-23
**P17987**	TCP1	69	629	31	31	0.000	1435.22	LNG
**P48643**	CCT5	74	752	38	37	0.000	1615.36	LNG
**P09493-4**	TPM1	54	343	21	4	0.000	485.07	LNG
**Q96CV9**	OPTN	15	26	6	6	0.000	56.06	EE + LNG, LNG, S-23
**P31949**	S100A11	50	173	5	5	0.000	320.86	EE + LNG, LNG
**P00450**	CP	21	74	17	17	0.000	126.06	LNG, S-23
**P00491**	PNP	78	207	20	20	0.000	487.30	LNG
**Q9H7Z6**	KAT8	6	8	3	2	0.000	8.98	EE + LNG, LNG, S-23
**Q9UHD8**	SEPTIN9	55	291	28	28	0.000	566.96	LNG, S-23
**P22102**	GART	60	552	46	46	0.000	1316.33	LNG
**O60264**	SMARCA5	47	416	55	38	0.000	713.32	LNG, S-23
**Q9NVA2**	SEPTIN11	48	226	16	9	0.000	484.63	LNG
**P12004**	PCNA	70	313	14	14	0.000	715.03	LNG, S-23
**Q13867**	BLMH	62	238	22	22	0.000	481.56	LNG
**P00492**	HPRT1	79	151	13	12	0.000	303.58	LNG
**P05121**	SERPINE1	27	46	8	8	0.000	90.21	EE + LNG, LNG, S-23
**Q9NZL9**	MAT2B	46	115	14	14	0.000	229.66	LNG
**O14529**	CUX2	0	6	1	1	0.044	5.16	LNG
**P48637**	GSS	61	204	23	23	0.000	425.83	LNG
**Q9NSY0**	NRBP2	22	50	7	7	0.000	109.32	LNG, S-23
**Q9UQ80**	PA2G4	60	504	23	23	0.000	964.01	LNG
**P42285**	MTREX	41	276	36	36	0.000	498.31	EE + LNG, LNG, S-23
**Q13491-3**	GPM6B	13	37	4	4	0.000	59.49	EE + LNG, LNG, S-23
**Q8TDB6**	DTX3L	25	50	13	13	0.000	112.25	LNG
**Q9UNH7**	SNX6	33	111	14	12	0.000	189.43	LNG
**Q9H4X1**	RGCC	8	4	1	1	0.011	6.57	LNG, S-23
**Q16270**	IGFBP7	10	12	2	2	0.000	19.20	EE + LNG, LNG, S-23
**P54886**	ALDH18A1	51	336	38	38	0.000	658.65	EE + LNG, LNG
**P08238**	HSP90AB1	59	2109	46	14	0.000	4334.71	LNG
**Q5UIP0**	RIF1	12	126	24	24	0.000	215.62	EE + LNG, LNG, S-23

Protein	Gene	Coverage	PSMs	Peptides	Unique Peptides	q-value	XCorr	Treatments
**P32455**	GBP1	16	27	7	7	0.000	42.74	LNG
**O43175**	PHGDH	52	331	22	22	0.000	882.43	LNG
**P00568**	AK1	75	142	14	14	0.000	258.77	LNG
**P17844**	DDX5	57	624	37	29	0.000	1036.72	LNG, S-23
**Q9P2R3**	ANKFY1	36	104	31	30	0.000	208.81	EE + LNG, LNG
**P33316**	DUT	47	162	10	10	0.000	312.12	EE + LNG, LNG, S-23
**P04181**	OAT	41	101	13	13	0.000	220.48	EE + LNG, LNG
**Q9GZL7**	WDR12	64	149	20	20	0.000	304.62	EE + LNG, LNG, S-23
**Q07960**	ARHGAP1	47	117	15	15	0.000	206.12	LNG
**Q9NRL2**	BAZ1A	21	107	28	28	0.000	184.91	LNG, S-23
**Q96CW1**	AP2M1	57	168	23	23	0.000	316.51	LNG
**Q9BQG0**	MYBBP1A	54	414	57	57	0.000	951.07	EE + LNG, LNG, S-23
**P39748**	FEN1	49	182	15	15	0.000	450.54	LNG, S-23
**P49748**	ACADVL	70	259	35	35	0.000	521.52	LNG, S-23
**P29320**	EPHA3	37	95	27	26	0.000	186.94	LNG
**Q99439**	CNN2	42	85	10	9	0.000	182.20	LNG
**P06493**	CDK1	67	161	17	15	0.000	335.23	LNG, S-23
**P07099**	EPHX1	58	167	23	23	0.000	363.86	EE + LNG, LNG, S-23
**O60506**	SYNCRIP	55	648	28	19	0.000	1371.30	LNG
**Q13492**	PICALM	36	159	17	17	0.000	279.49	EE + LNG, LNG
**Q12788**	TBL3	60	206	34	34	0.000	475.91	EE + LNG, LNG
**P61221**	ABCE1	52	279	30	30	0.000	574.60	EE + LNG, LNG
**P13196**	ALAS1	5	12	3	3	0.000	13.03	EE + LNG, LNG, S-23
**Q01780**	EXOSC10	46	192	34	34	0.000	360.82	LNG
**Q9P035**	HACD3	32	156	12	12	0.000	343.14	LNG
**Q6DKJ4**	NXN	36	55	9	9	0.000	143.69	LNG
**Q9Y3A2**	UTP11	35	47	9	9	0.000	77.06	EE + LNG, LNG
**P00966**	ASS1	53	213	17	17	0.000	469.23	EE + LNG
**P07237**	P4HB	61	588	33	33	0.000	1047.74	EE + LNG
**Q15075**	EEA1	45	281	58	58	0.000	508.09	EE + LNG, S-23
**P27708**	CAD	56	759	87	87	0.000	1804.78	EE + LNG
**P08243**	ASNS	55	284	24	24	0.000	451.83	EE + LNG
**P49588**	AARS1	64	645	49	49	0.000	1381.43	EE + LNG
**Q92823**	NRCAM	26	124	26	26	0.000	258.34	EE + LNG, S-23
**P42704**	LRPPRC	71	977	79	79	0.000	1959.70	EE + LNG
**Q7KZF4**	SND1	64	636	46	46	0.000	1265.29	EE + LNG
**P23381**	WARS1	53	191	21	21	0.000	400.98	EE + LNG
**O43707**	ACTN4	80	1260	69	43	0.000	2769.18	EE + LNG, S-23

Protein	Gene	Coverage	PSMs	Peptides	Unique Peptides	q-value	XCorr	Treatments
**P49591**	SARS1	53	289	24	24	0.000	693.04	EE + LNG
**Q13683-7**	ITGA7	37	194	30	30	0.000	455.01	EE + LNG
**P26038**	MSN	67	1014	49	33	0.000	1875.92	EE + LNG
**O75083**	WDR1	71	380	30	30	0.000	928.05	EE + LNG
**P17174**	GOT1	67	160	20	20	0.000	314.58	EE + LNG
**P23368**	ME2	50	188	22	21	0.000	421.44	EE + LNG
**P13489**	RNH1	73	167	23	23	0.000	573.54	EE + LNG
**Q9HAV4**	XPO5	40	288	37	37	0.000	688.93	EE + LNG, S-23
**P13473**	LAMP2	15	60	6	6	0.000	108.32	EE + LNG
**P08240**	SRPRA	57	195	28	28	0.000	358.65	EE + LNG
**Q07065**	CKAP4	73	522	45	44	0.000	930.86	EE + LNG
**P52943**	CRIP2	20	55	3	3	0.000	118.55	EE + LNG
**Q6UX53**	TMT1B	53	151	10	10	0.000	301.59	EE + LNG
**Q16836**	HADH	46	183	12	12	0.000	320.31	EE + LNG
**P17677**	GAP43	80	161	17	17	0.000	434.34	EE + LNG
**O14933**	UBE2L6	37	15	4	4	0.000	28.56	EE + LNG, S-23
**P30405**	PPIF	45	92	8	6	0.000	193.56	EE + LNG
**P51178**	PLCD1	27	56	12	12	0.000	149.42	EE + LNG, S-23
**Q06203**	PPAT	33	93	15	15	0.000	169.10	EE + LNG
**P43246**	MSH2	41	252	30	30	0.000	443.11	EE + LNG, S-23
**P55809**	OXCT1	43	100	16	16	0.000	226.69	EE + LNG
**O00566**	MPHOSPH10	29	86	16	16	0.000	154.01	EE + LNG
**Q9NU22**	MDN1	15	230	63	63	0.000	439.76	EE + LNG, S-23
**Q9Y4B6**	DCAF1	13	63	17	17	0.000	89.83	EE + LNG, S-23
**Q9UQE7**	SMC3	46	329	49	49	0.000	619.87	EE + LNG, S-23
**Q16363**		1	8	1	1	0.005	12.41	EE + LNG
**Q9H299**	SH3BGRL3	52	101	6	6	0.000	167.66	EE + LNG
**Q9H2P0**	ADNP	24	123	22	22	0.000	228.28	EE + LNG, S-23
**P40121**	CAPG	27	38	7	7	0.000	73.63	EE + LNG
**P60709**	ACTB	76	3342	29	11	0.000	8264.85	EE + LNG
**P11021**	HSPA5	59	1275	40	37	0.000	2454.87	EE + LNG
**Q9UP95**	SLC12A4	15	72	13	12	0.000	127.24	EE + LNG
**Q14692**	BMS1	27	158	28	28	0.000	289.98	EE + LNG
**P12955**	PEPD	49	109	17	17	0.000	281.26	EE + LNG, S-23
**Q9BXW9-1**	FANCD2	16	60	18	18	0.000	99.81	EE + LNG, S-23
**Q15269**	PWP2	40	193	30	30	0.000	364.51	EE + LNG, S-23
**Q96KB5**	PBK	39	70	11	11	0.000	111.18	EE + LNG, S-23
**Q9H8H0**	NOL11	50	130	24	24	0.000	266.72	EE + LNG

Protein	Gene	Coverage	PSMs	Peptides	Unique Peptides	q-value	XCorr	Treatments
**Q8NI36**	WDR36	28	100	22	22	0.000	169.96	EE + LNG, S-23
**Q96TA1**	NIBAN2	46	365	28	28	0.000	743.38	EE + LNG
**P07951-2**	TPM2	56	394	23	2	0.000	597.00	EE + LNG
**Q13557-10**	CAMK2D	56	323	22	1	0.000	656.66	EE + LNG
**Q9Y5K5**	UCHL5	49	87	12	12	0.000	167.50	EE + LNG
**Q96SB4**	SRPK1	26	90	16	13	0.000	174.99	EE + LNG
**O75691**	UTP20	27	307	65	65	0.000	566.16	EE + LNG
**Q9H501**	ESF1	23	74	16	16	0.000	120.57	EE + LNG
**P11388**	TOP2A	41	420	57	40	0.000	689.30	S-23
**P52732**	KIF11	26	104	22	22	0.000	191.26	S-23
**P18858**	LIG1	20	81	12	12	0.000	166.84	S-23
**P06737**	PYGL	61	488	50	40	0.000	838.33	S-23
**Q96RT1**	ERBIN	21	117	25	25	0.000	180.06	S-23
**Q9NRZ9**	HELLS	16	36	11	11	0.000	52.18	S-23
**P13987**	CD59	25	20	3	3	0.000	50.53	S-23
**P31689**	DNAJA1	63	264	20	20	0.000	612.58	S-23
**P52292**	KPNA2	45	151	19	19	0.000	370.66	S-23
**Q13257**	MAD2L1	52	86	10	10	0.000	146.48	S-23
**Q14683**	SMC1A	51	397	60	60	0.000	660.89	S-23
**P13861**	PRKAR2A	57	150	19	17	0.000	251.56	S-23
**O95239**	KIF4A	23	120	24	23	0.000	210.99	S-23
**Q9Y287**	ITM2B	37	45	6	6	0.000	79.89	S-23
**P27105**	STOM	47	56	10	10	0.000	110.81	S-23
**O75330**	HMMR	16	33	9	8	0.000	66.23	S-23
**Q9NS87**	KIF15	16	69	21	21	0.000	101.21	S-23
**Q15397**	PUM3	42	138	21	21	0.000	274.52	S-23
**Q9BZE4**	GTPBP4	41	169	24	24	0.000	311.92	S-23
**Q92922**	SMARCC1	28	167	25	16	0.000	281.24	S-23
**P00374**	DHFR	69	116	12	12	0.000	169.59	S-23
**Q9BPX3**	NCAPG	34	138	27	27	0.000	275.54	S-23
**Q9Y6X5**	ENPP4	18	26	6	6	0.000	62.13	S-23
**P52701**	MSH6	40	236	42	42	0.000	462.93	S-23
**Q96T88**	UHRF1	55	236	32	31	0.000	494.68	S-23
**P49642**	PRIM1	28	33	9	9	0.000	58.33	S-23
**P22570**	FDXR	60	133	23	23	0.000	245.80	S-23
**Q9Y2D5-6**	PALM2AKAP2	16	77	15	13	0.000	143.78	S-23
**P35249**	RFC4	61	100	16	16	0.000	236.96	S-23
**Q15398**	DLGAP5	6	10	4	4	0.000	19.80	S-23

Protein	Gene	Coverage	PSMs	Peptides	Unique Peptides	q-value	XCorr	Treatments
**P04818**	TYMS	43	69	9	9	0.000	132.22	S-23
**P19823**	ITIH2	1	4	1	1	0.058	0.00	S-23
**O94776**	MTA2	54	241	32	27	0.000	375.97	S-23
**P09525**	ANXA4	69	170	20	19	0.000	234.94	S-23
**Q9NZN4**	EHD2	43	131	17	14	0.000	251.66	S-23
**Q9NVP2**	ASF1B	18	14	3	2	0.000	23.66	S-23
**Q9HCS7**	XAB2	32	100	18	18	0.000	217.10	S-23
**P31350**	RRM2	54	76	18	16	0.000	135.36	S-23
**Q99729-3**	HNRNPAB	53	312	18	16	0.000	588.61	S-23
**Q15003**	NCAPH	29	111	16	16	0.000	222.59	S-23
**Q765P7**	MTSS2	15	39	8	8	0.000	83.61	S-23
**P06400**	RB1	29	156	25	25	0.000	279.17	S-23
**Q13823**	GNL2	28	79	17	17	0.000	139.18	S-23
**P10619**	CTSA	24	63	10	10	0.000	142.12	S-23
**Q8N3U4**	STAG2	27	183	28	22	0.000	291.38	S-23
**O95235**	KIF20A	10	37	7	7	0.000	65.37	S-23
**Q15392**	DHCR24	38	154	17	17	0.000	324.84	S-23
**Q9UGN5**	PARP2	21	37	10	10	0.000	64.51	S-23
**Q15645**	TRIP13	53	122	20	20	0.000	219.04	S-23

Enriched GO terms

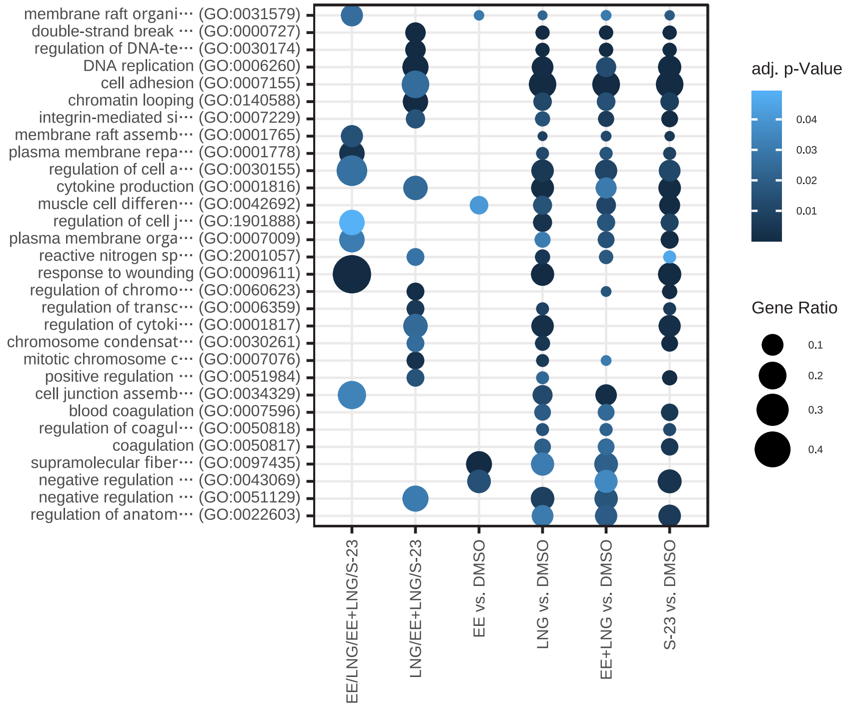

Figure S9 Enriched GO terms.
Bubble plot showing the top 30 GO-enriched biological processes across different experimental contrasts and their intersections. Each row represents a GO term, while columns represent specific pairwise comparisons: EE/LNG/EE + LNG/S-23, LNG/EE+LNG/S-23, EE vs. DMSO, LNG vs. DMSO, EE + LNG vs. DMSO, and S-23 vs. DMSO. The size of each bubble reflects the gene ratio, and the color intensity indicates the adjusted p-value, with darker shades representing stronger statistical significance. This visualization highlights shared and unique biological processes affected under various treatment conditions.
(Identical to Figure 5 of the manuscript.)

Table 4 Top 30 most significantly enriched GO terms.

ID	Description
**GO:0031579**	membrane raft organization
**GO:0000727**	double-strand break repair via break-induced replication
**GO:0030174**	regulation of DNA-templated DNA replication initiation
**GO:0006260**	DNA replication
**GO:0007155**	cell adhesion
**GO:0140588**	chromatin looping
**GO:0007229**	integrin-mediated signaling pathway
**GO:0001765**	membrane raft assembly
**GO:0001778**	plasma membrane repair
**GO:0030155**	regulation of cell adhesion
**GO:0001816**	cytokine production
**GO:0042692**	muscle cell differentiation
**GO:1901888**	regulation of cell junction assembly
**GO:0007009**	plasma membrane organization
**GO:2001057**	reactive nitrogen species metabolic process
**GO:0009611**	response to wounding
**GO:0060623**	regulation of chromosome condensation
**GO:0006359**	regulation of transcription by RNA polymerase III
**GO:0001817**	regulation of cytokine production
**GO:0030261**	chromosome condensation
**GO:0007076**	mitotic chromosome condensation
**GO:0051984**	positive regulation of chromosome segregation
**GO:0034329**	cell junction assembly
**GO:0007596**	blood coagulation
**GO:0050818**	regulation of coagulation
**GO:0050817**	coagulation
**GO:0097435**	supramolecular fiber organization
**GO:0043069**	negative regulation of programmed cell death
**GO:0051129**	negative regulation of cellular component organization
**GO:0022603**	regulation of anatomical structure morphogenesis

Enriched KEGG pathways

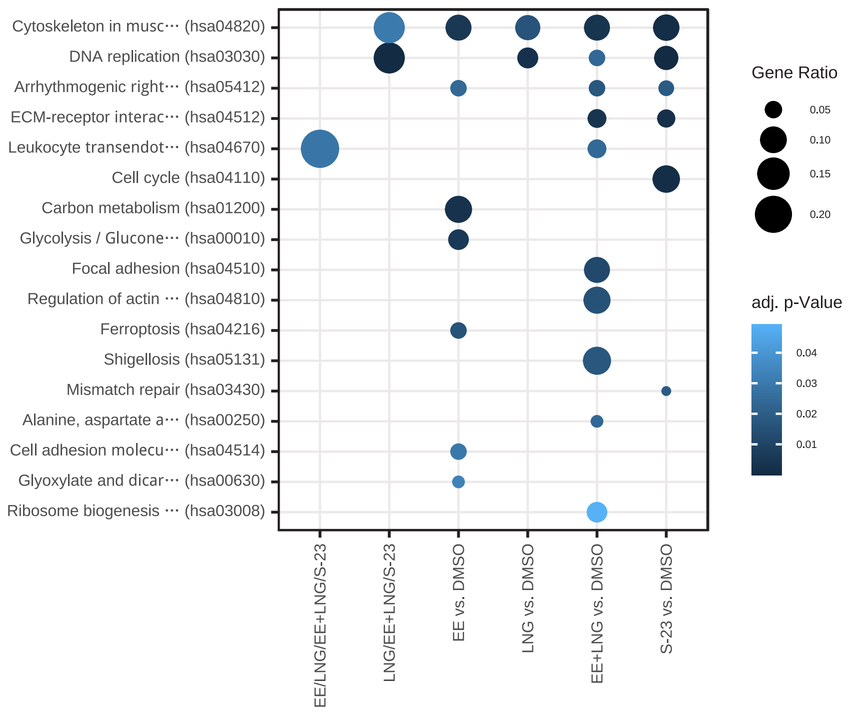

Figure S10 Enriched KEGG terms.

Bubble plot showing the KEGG-enriched pathways across different experimental contrasts and their intersections. Each row represents a KEGG pathway, while columns represent specific pairwise comparisons: EE/LNG/EE + LNG/S-23, LNG/EE+LNG/S-23, EE vs. DMSO, LNG vs. DMSO, EE + LNG vs. DMSO, and S-23 vs. DMSO. The size of each bubble reflects the gene ratio, and the color intensity indicates the adjusted p-value, with darker shades representing stronger statistical significance. This visualization highlights shared and unique pathways affected under various treatment conditions.

Table S5 All the enriched KEGG pathways.

ID	Description
**hsa04820**	Cytoskeleton in muscle cells
**hsa03030**	DNA replication
**hsa05412**	Arrhythmogenic right ventricular cardiomyopathy
**hsa04512**	ECM-receptor interaction
**hsa04670**	Leukocyte transendothelial migration
**hsa04110**	Cell cycle
**hsa01200**	Carbon metabolism
**hsa00010**	Glycolysis / Gluconeogenesis
**hsa04510**	Focal adhesion
**hsa04810**	Regulation of actin cytoskeleton
**hsa04216**	Ferroptosis
**hsa05131**	Shigellosis
**hsa03430**	Mismatch repair
**hsa00250**	Alanine, aspartate and glutamate metabolism
**hsa04514**	Cell adhesion molecules
**hsa00630**	Glyoxylate and dicarboxylate metabolism
**hsa03008**	Ribosome biogenesis in eukaryotes

Enriched DOSE terms

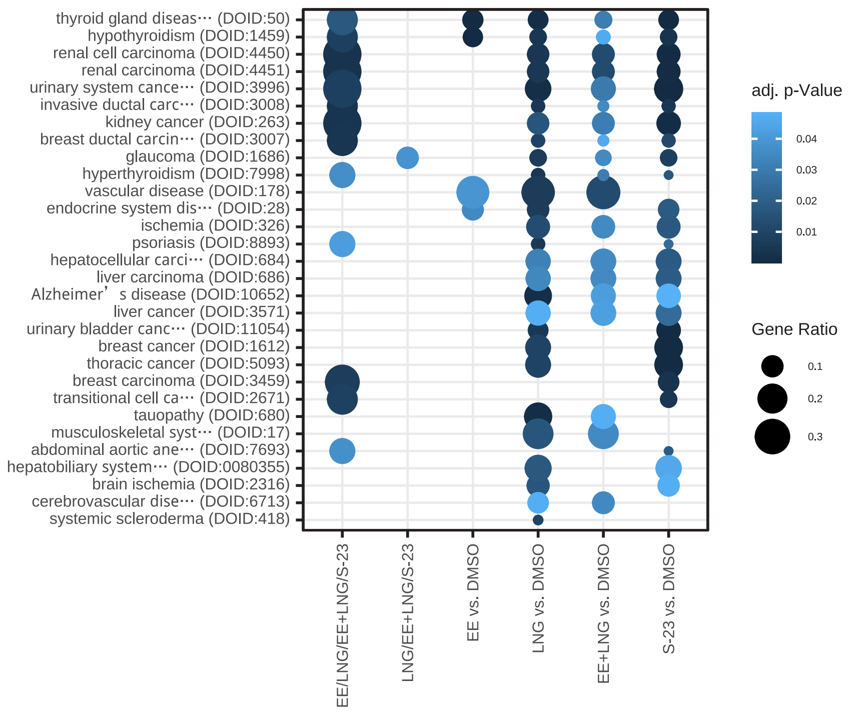

Figure S11 Enriched DOSE terms.

Bubble plot showing the top 30 DOSE-enriched terms across different experimental contrasts and their intersections. Each row represents a DOSE term, while columns represent specific pairwise comparisons: EE/LNG/EE + LNG/S-23, LNG/EE+LNG/S-23, EE vs. DMSO, LNG vs. DMSO, EE + LNG vs. DMSO, and S-23 vs. DMSO. The size of each bubble reflects the gene ratio, and the color intensity indicates the adjusted p-value, with darker shades representing stronger statistical significance. This visualization highlights shared and unique diseases associated to various treatment conditions.

Table S6 Top 30 most significantly enriched DOSE terms.

ID	Description
**DOID:50**	thyroid gland disease
**DOID:1459**	hypothyroidism
**DOID:4450**	renal cell carcinoma
**DOID:4451**	renal carcinoma
**DOID:3996**	urinary system cancer
**DOID:3008**	invasive ductal carcinoma
**DOID:263**	kidney cancer
**DOID:3007**	breast ductal carcinoma
**DOID:1686**	glaucoma
**DOID:7998**	hyperthyroidism
**DOID:178**	vascular disease
**DOID:28**	endocrine system disease
**DOID:326**	ischemia
**DOID:8893**	psoriasis
**DOID:684**	hepatocellular carcinoma
**DOID:686**	liver carcinoma
**DOID:10652**	Alzheimer's disease
**DOID:3571**	liver cancer
**DOID:11054**	urinary bladder cancer
**DOID:1612**	breast cancer
**DOID:5093**	thoracic cancer
**DOID:3459**	breast carcinoma
**DOID:2671**	transitional cell carcinoma
**DOID:680**	tauopathy
**DOID:17**	musculoskeletal system disease
**DOID:7693**	abdominal aortic aneurysm
**DOID:0080355**	hepatobiliary system cancer
**DOID:2316**	brain ischemia
**DOID:6713**	cerebrovascular disease
**DOID:418**	systemic scleroderma

Enriched DisGeNET terms

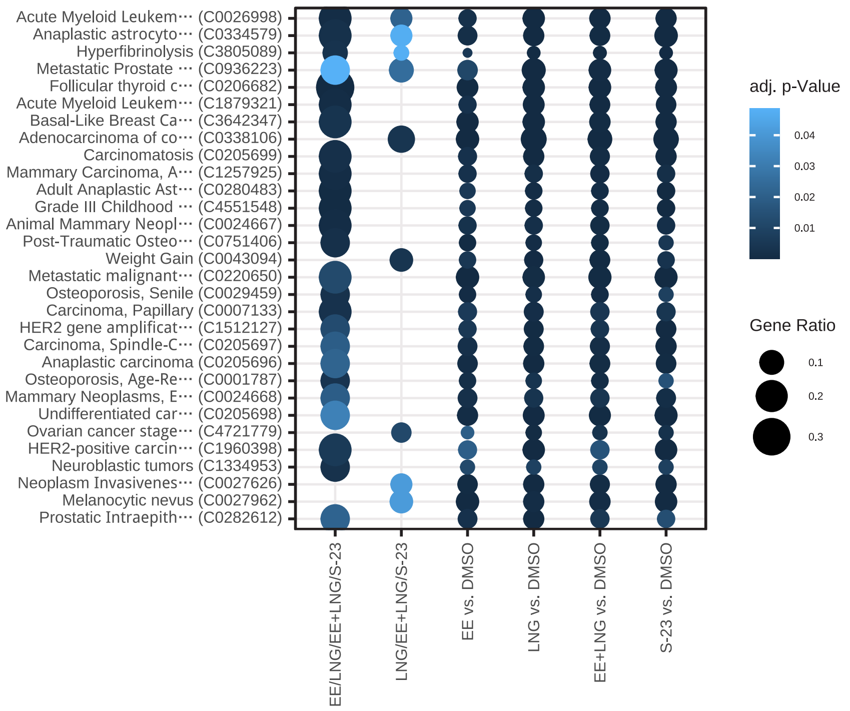

Figure S12 Enriched DisGeNET entries.
Bubble plot showing the top 30 DisGeNET-enriched entries across different experimental contrasts and their intersections. Each row represents a DisGeNET entry, while columns represent specific pairwise comparisons: EE/LNG/EE + LNG/S-23, LNG/EE+LNG/S-23, EE vs. DMSO, LNG vs. DMSO, EE + LNG vs. DMSO, and S-23 vs. DMSO. The size of each bubble reflects the gene ratio, and the color intensity indicates the adjusted p-value, with darker shades representing stronger statistical significance. This visualization highlights shared and unique diseases associated to various treatment conditions.

Table S7 Top 30 most significantly enriched DisGeNET entries.

ID	Description
**C0026998**	Acute Myeloid Leukemia, M1
**C0334579**	Anaplastic astrocytoma
**C3805089**	Hyperfibrinolysis
**C0936223**	Metastatic Prostate Carcinoma
**C0206682**	Follicular thyroid carcinoma
**C1879321**	Acute Myeloid Leukemia (AML-M2)
**C3642347**	Basal-Like Breast Carcinoma
**C0338106**	Adenocarcinoma of colon
**C0205699**	Carcinomatosis
**C1257925**	Mammary Carcinoma, Animal
**C0280483**	Adult Anaplastic Astrocytoma
**C4551548**	Grade III Childhood Astrocytoma
**C0024667**	Animal Mammary Neoplasms
**C0751406**	Post-Traumatic Osteoporosis
**C0043094**	Weight Gain
**C0220650**	Metastatic malignant neoplasm to brain
**C0029459**	Osteoporosis, Senile
**C0007133**	Carcinoma, Papillary
**C1512127**	HER2 gene amplification
**C0205697**	Carcinoma, Spindle-Cell
**C0205696**	Anaplastic carcinoma
**C0001787**	Osteoporosis, Age-Related
**C0024668**	Mammary Neoplasms, Experimental
**C0205698**	Undifferentiated carcinoma
**C4721779**	Ovarian cancer stage IV
**C1960398**	HER2-positive carcinoma of breast
**C1334953**	Neuroblastic tumors
**C0027626**	Neoplasm Invasiveness
**C0027962**	Melanocytic nevus
**C0282612**	Prostatic Intraepithelial Neoplasias
